# Extreme Coastal Water Levels Exacerbate Fluvial Flood Hazards in Northwestern Europe

**DOI:** 10.1038/s41598-019-49822-6

**Published:** 2019-09-11

**Authors:** Poulomi Ganguli, Bruno Merz

**Affiliations:** 1 Agricultural and Food Engineering Department, Indian Institute of Technology Kharagpur, Kharagpur, India; 2 Helmholtz Centre Potsdam, GFZ German Research Centre for Geosciences, Potsdam, Germany; 3 Institute of Environmental Sciences and Geography, University of Potsdam, Potsdam, Germany

**Keywords:** Hydrology, Natural hazards

## Abstract

Compound flooding, such as the co-occurrence of fluvial floods and extreme coastal water levels (CWL), may lead to significant impacts in densely-populated Low Elevation Coastal Zones. They may overstrain disaster management owing to the co-occurrence of inundation from rivers and the sea. Recent studies are limited by analyzing joint dependence between river discharge and either CWL or storm surges, and little is known about return levels of compound flooding, accounting for the covariance between drivers. Here, we assess the compound flood severity and identify hotspots for northwestern Europe during 1970–2014, using a newly developed Compound Hazard Ratio (CHR) that compares the severity of compound flooding associated with extreme CWL with the unconditional *T*-year fluvial peak discharge. We show that extreme CWL and stronger storms greatly amplify fluvial flood hazards. Our results, based on frequency analyses of observational records during 2013/2014’s winter storm Xaver, reveal that the river discharge of the 50*-*year compound flood is up to 70% larger, conditioned on the occurrence of extreme CWL, than that of the at-site peak discharge. For this event, nearly half of the stream gauges show increased flood hazards, demonstrating the importance of including the compounding effect of extreme CWL in river flood risk management.

## Introduction

In 2000, ~ 33 million people lived in Low Elevation Coastal Zones (LECZ) in northwestern Europe^1^. The North Sea is one of the most industrial seas in the world, providing an estimated gross added value of €150 billion to the surrounding European countries^2^. Flood hazard in LECZ is rarely a function of one process alone but comprises multiple drivers, including river discharge and extreme Coastal Water Levels^3–6^ (CWL). The latter includes both tidal (high-tide flooding) and non-tidal effects (surges). Between 1961 and 2017, two of the coastal nations in northwestern Europe, Sweden and the Netherlands, have experienced ~ 0.74% and 0.21% shrinkage in total land masses^7,8^ due to flood-induced coastal erosion and sea level rise^9,10^. The interactions between fluvial and coastal processes have begun to attract attention due to the flood vulnerability of river deltas^11,12^. As sea level rise pushes ocean tides upstream^13^, the tidal signal propagates from river estuaries to inland leading to a gradual shift from non-tidal to tidally influenced river flow. Subsurface resources exploitation and soil compaction by urban growth lead to deltaic subsidence and aggravate the interactions between the river and coastal processes in many river deltas globally^14,15^.

Coastal flood hazard assessments are typically based on univariate approaches assuming distributions to be stationary and unconditional, either fluvial floods^16^ or extreme sea levels (or storm surges^17^). Nevertheless, using physically-based and stochastic models, a few local studies^6,18,19^ have explored the influence of compound events on flood hazards. Sayol and Marcos^20^ analysed the compound effects of the surges and waves over the Ebro Delta, Spain. Hawkes *et al*.^21^ investigated simultaneous occurrences of large waves and a high CWL along the North Sea and the Irish Sea. However, in these studies, the interactions with river floods were not considered. River discharge can influence coastal ocean circulation affecting sea-level change, and often act as a driver of coastal flood risk^22^. Tessler *et al*.^12^ quantified changes in flood risk at 48 major deltas, however, no distinction was made for the relative contribution of fluvial or coastal floods in estimating the hazard. Most efforts on compound flooding to date are limited to either analysing dependence among multiple drivers^23–25^ or determining bivariate joint probability and/or joint return periods^3,4,26–29^, and do not provide information about the likelihood and intensity of fluvial floods conditional on CWL. Paprotny *et al*.^28^ derived Europe-wide compound flood indices, analysing co-occurrence of storm surge with either the 10-year precipitation (as a proxy for flash floods) or the 10-year river discharge simulated by a large-scale hydrological model (as a proxy for river flood). However, these indices do not take into account extreme CWL^30,31^ resulting from both tidal (i.e., high tide flooding) and non-tidal processes.

While it has been argued that stronger dependence between different drivers increases the risk of compound floods, to our knowledge, most efforts limit their analysis to interdependencies between the meteorological drivers, storm surge and heavy precipitation^3,26,27^, assuming the latter as a proxy for fluvial floods. However, heavy precipitation does not necessarily lead to fluvial floods; the response of the affected catchment to precipitation depends strongly on a range of factors, such as the antecedent catchment wetness^32,33^. Analysing 32 European river mouths, Petroliagkis *et al*.^34^ found that a lag-time of a few days was required to establish a moderate to a strong correlation between surge and river discharge. Using kinematic wave-based formulations^35^ of bankfull flow-time estimates and wave celerity (the velocity at which large waves propagate downstream), Ward *et al*.^36^ estimated average flow-times between stream gauges and coastal catchment outlets. However, global estimates of flow time are not available for most of Scandinavia (>60°N).

Our analyses fill existing gaps in the literature in multiple ways. First, we demonstrate spatial patterns of correlations between extreme CWL resulting from tidal and non-tidal processes and peak river discharge over 500 pairs of tidal (TG) – stream gauges (SG) along the North Sea coast. Extreme CWL primarily results from the combination of various factors such as astronomical tides and a large-scale rise of the sea surface caused by high wind speeds and low atmospheric pressure^37–39^; however, in many areas storm-driven residuals are often weak and impacts are larger when surges coincide with high spring tides. Haigh *et al*.^40^ showed that the majority of extreme sea level events along the European coast were generated by moderate, rather than extreme skew surges, combined with spring astronomical high tides. For the North Sea and the English Channel, an interaction between storm surges and tides has been shown as the maximum skew surges are more likely to occur 3–5 hours before (i.e., at rising tide) tidal high water, which can amplify surge magnitude^41,42^.

**Figure 1:**
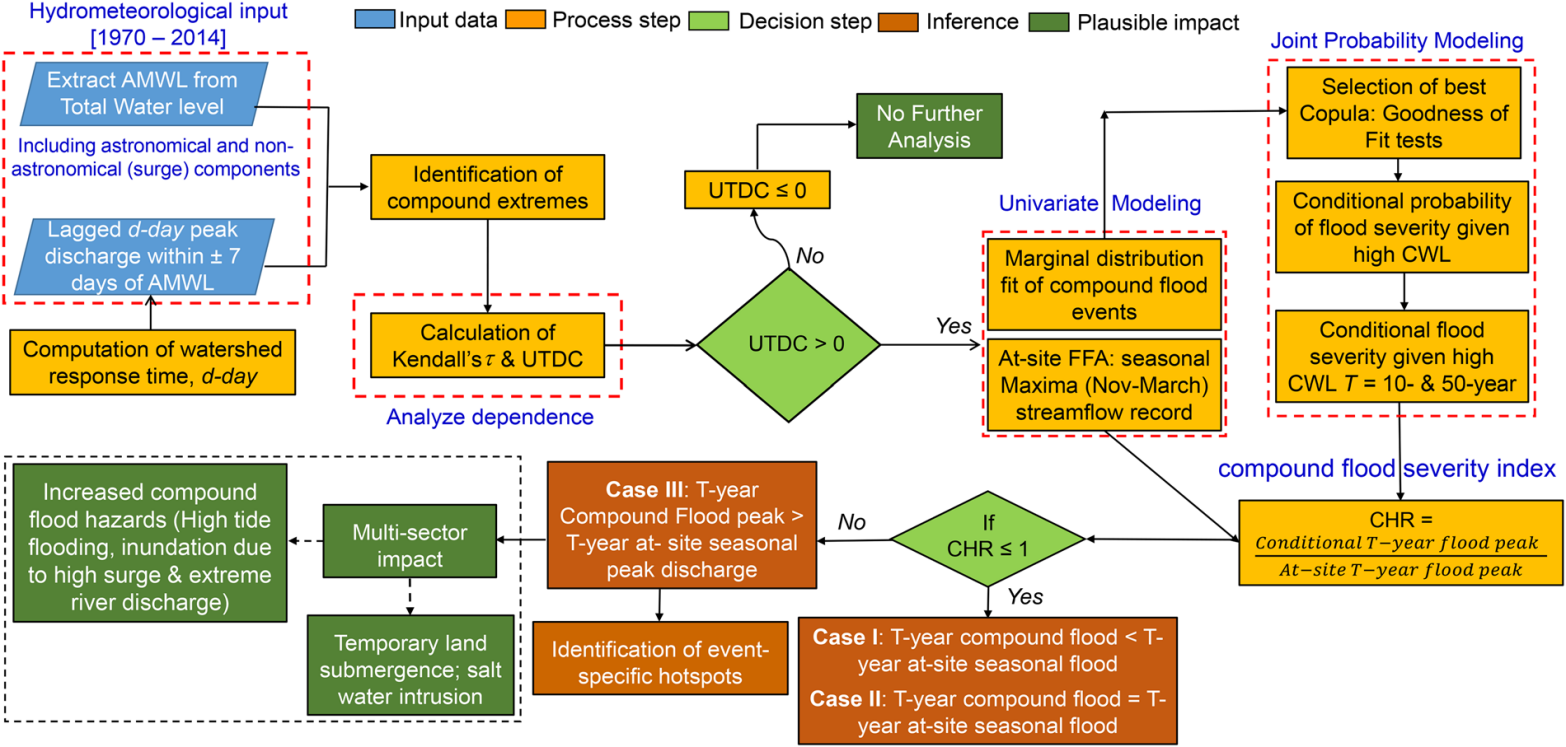
Workflow for analyses of compound flooding and Identification of hotspots; FFA, CHR, UTDC, CWL, AMWL, and *T* denote Flood Frequency Analysis, Compound Hazard Ratio, Upper Tail Dependence Coefficients, Coastal Water Level, Annual Maxima Water Level, and return period, respectively. The flowchart is prepared in MS Office Power Point 2010 and then organized in Adobe Photoshop CS6 Desktop (Version 13.0.1 × 32, http://www.adobe.com) [Software].

We define compound floods as a result of two distinct mechanisms: (1) Extreme coastal water levels (CWL) may affect river flows and water levels by backwater effects or by reversing the seaward flow of river. The tidal signal may propagate as much as over 500 km inland, increasing flood risk far from the coast^11^. Rivers in the regions with elevation less than 10 m in northwestern Europe (Fig. 1 in Hoitink and Jay^11^) are likely to be influenced by this mechanism. (2) The correlation between high CWL and fluvial peak discharge may also stem from a common meteorological cause. Severe storm periods may be associated with high winds leading to storm surges, and at the same time with high precipitation followed by inland flooding^3^. Following previous studies, we consider (hourly) annual maxima of total CWL^4–6,21,36,43–45^ measured by the TG and corresponding peak river discharge as major flood drivers. Annual maxima of total water level typically composed of three elements, astronomical tide height, mean sea level and non-tidal residual components that captures the effects of storm surges, inter-annual variability such as El-Nino and other processes. The coastal ocean forms the downstream boundary of a river that causes both river stage and flow discharge to be influenced by all three elements, i.e. the total coastal water level^11^. On the other hand, increased river discharge during flood events not only raise mean coastal water levels but also works as a friction component that makes tides lose energy and shrink in amplitude^22,46^. Also, Sassi and Hoitink^47^ have shown that even for high river flow and low-tidal velocity amplitudes, river-tide interaction contributes significantly to subtidal friction, impacting river discharge downstream. The non-tidal residuals can be considered a stochastic process, which is driven by meteorological conditions. Further, both mean sea level and tides show seasonal to decadal variability in addition to long-term trends^48^. The hourly extreme water level provided by TG takes into account tidal and non-tidal processes (such as skew surge and non-tidal residuals), whereas wave effects do not affect water level observations^39,43^, because TGs are typically located in sheltered locations that limit direct impact of winds relative to open coastlines. The TGs are carefully selected such that the longest and highest-quality data are analysed. Since the large astronomical tidal variabilities could mask the correlation between non-tidal processes and fluvial discharge^4^, the nonlinear interaction between extreme CWL and fluvial peak discharge is analysed using an array of non-parametric dependence metrics^49^.

Second, earlier literature dealt with compound flooding from high coastal water level (or surge) and rainfall-driven floods in the North America^26^, Australia^27^ and South China^29^. Likewise, Kew *et al*.^3^ assessed compound effects of North Sea storm surges and extreme Rhine river discharge for the current and projected climate in a large 17-member global climate model ensemble. They have used north-northwesterly winds over the North Sea as a proxy for the storm surge and multiple-day precipitation (up to 20-day lag) over the Rhine basin as the proxy for the River discharge. Bevacqua *et al*.^44^ have assessed the compound flood hazards of European coasts resulting from the co-occurrence of extreme sea levels (i.e., daily maximum values of the superposition of surges, including waves and astronomical tides) and heavy precipitation for present (1970–2004) and the projected (2070–2099) climate considering the business-as-usual (RCP8.5) scenario. A few studies have investigated compound flood hazards from surge and river floods on the European coast. Klerk *et al*.^50^ employed the Delft Continental Shelf Model to simulate high storm surge levels at Hoek van Holland and a lumped hydrologic model (HBV) to simulate daily river runoff of the Rhine River at the German-Dutch border. Dependence was found to be highest for a six days’ time lag, which was introduced through the HBV hydrological model that accounts for the antecedent water storage within the basin and the delay between rainfall and streamflow. However, their assessment was limited to one river gauge only. Paprotny *et al*.^28^ analysed surge simulated through Delft3D and daily river discharge using a distributed hydrological model (LISFLOOD) driven by the gridded meteorological forcing data with a time lag of up to + 3 days. In most of the assessments^4,5,25,26,29,44^ dependencies between the two variables were analysed at a time lag of zero to ± 1-days; the delay between the rainfall and the streamflow^51^ was not considered. We take into account the response time of catchments^52–54^ (Methods) to storm events, which was ignored in most earlier assessments. In estuarine regions, compound flooding can occur from the superposition of a severe storm resulting from a suite of meteorological drivers (such as extreme wind and persistent heavy precipitation), that causes an extreme CWL and a peak discharge that travels along the river to the coast. While both, seen in isolation, may not have a significant impact, their coincidence or successive occurrences of both events may exacerbate the impact of flooding in the coastal area.

Third, we quantify the severity of compound floods at individual stream gauge locations by developing a dimensionless index, the Compound Hazard Ratio (CHR; see Methods), particularly valuable for analyzing compound hazards and communicate risks to a broader audience. It considers the effect of a physical covariate (i.e., extreme CWL) and compares the severity of river floods conditional on extreme CWL with at-site, unconditional *T*-year peak discharge. Identifying event-specific hotspots or the set of river gauges that have increased probabilities of flooding when the coast is hit by a severe storm is highly relevant information for flood risk management. A strong dependence between high CWL and high river discharge could lead to situations where the disaster management capacities are more easily overtaxed since not only the coast but also inland rivers might show inundation. However, it should be noted that we do not evaluate associated impacts such as inundation areas and damage. Finally, in our analyses we made a significant effort to ensure that the selected extremes, i.e. annual maxima extreme CWLs, are independent and identically distributed, an aspect not thoroughly considered in the previous assessments^3,19,50,55^.

Our modelling framework, described in Fig. 1, identifies compound flooding hotspots and allows a robust assessment of the associated hazards.

## Results

This paper develops a dimensionless index to quantify compound flooding (i.e., the coincidence of HCWL and peak discharge). We demonstrated the applicability of the methodology in assessing the severity of compound flood hazard along the northwestern European coastline through three catastrophic storm episodes. We select northwestern Europe as the test bed since the region is extremely vulnerable to severe storm-induced compound flooding^36,56–58^.

### Spatial variability in extreme coastal water level-peak flow dependence

**Figure 2:**
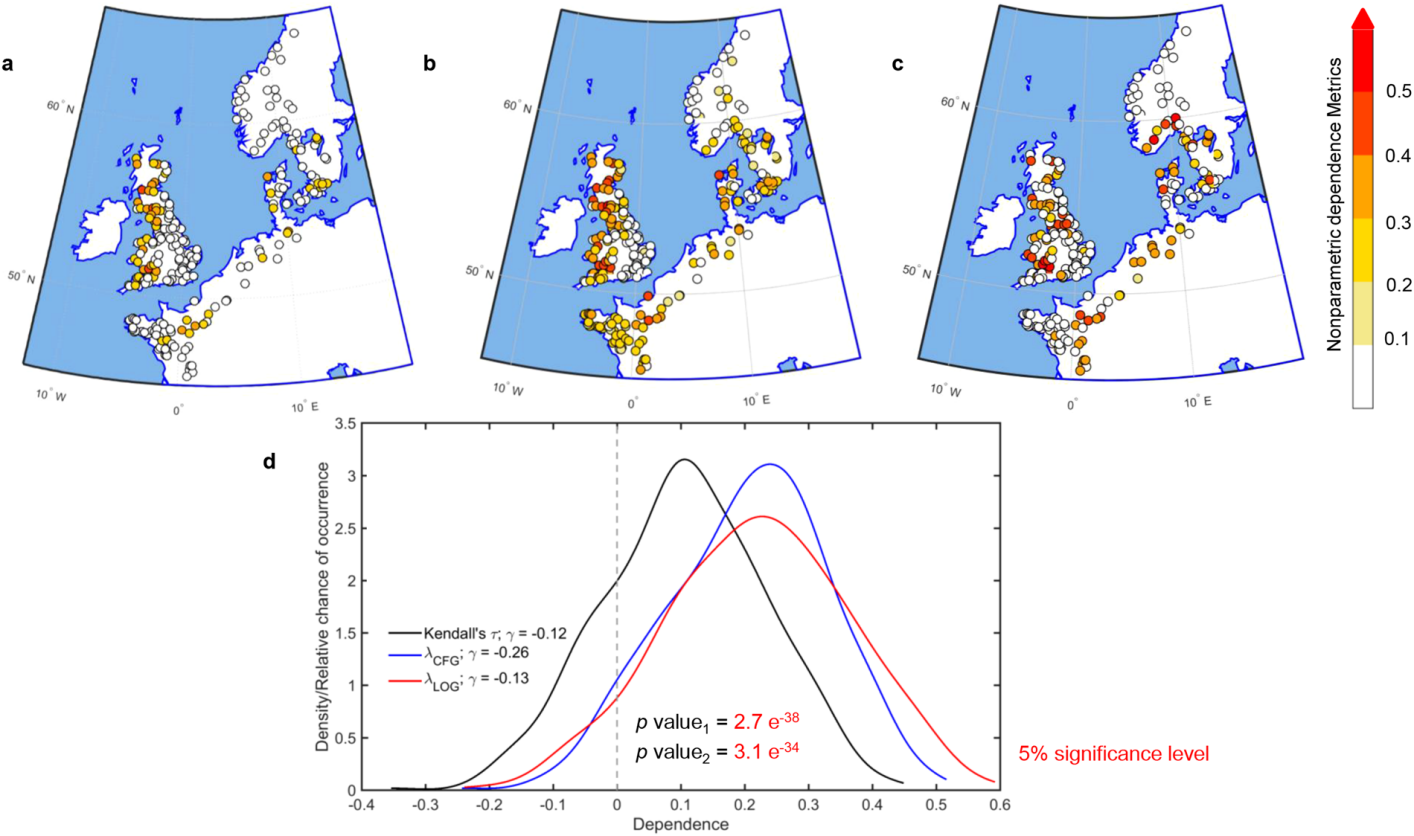
Dependence between extreme CWL and river peak discharge. (a-c) Spatial maps of correlation and upper tail dependence between annual maxima CWL and *d-day* lagged daily peak discharge within ± 7 days of the occurrence of the extreme CWL using nonparametric dependence measures. The complete dependence between two variables is established using Kendall’s τ (**a**), while the Upper Tail Dependence Coefficients, UTDC (**b**,**c**), are computed using two nonparametric upper tail dependence metrics (see Methods). The location of SGs with significant (at 5% level) dependence between compound flood drivers are marked with colours, whereas the location of SGs with insignificant and low values of positive dependence (with values <0.1 and p-values ≥0.05), and negative dependence (values <0) are marked with white (**d**) Kernel density functions of complete and UTDC metrics illustrating the negative skewness in the spatial distribution. The two UTDC distributions are shifted significantly (as indicated by the *p-*values < 0.05) towards higher values relative to the distribution of complete dependence. The density curve in LOG estimator is flattened, with an elongated right (higher dependence) tail. Maps are generated using MATLAB 2015a (Version 8.5, http://www.mathworks.com), and then organized and labelled in Adobe Photoshop CS6 Desktop (Version 13.0.1 × 32, http://www.adobe.com) [Software].

We quantify the strength of dependence between CWLs and river peak flows using complete and upper tail dependence metrics (Fig. 2). We find spatially coherent patterns across the different metrics, such as strong positive dependence along the French Coast, western UK, Denmark and Sweden, and weak positive dependencies along the northern coast of Norway (Fig. 2a–c). On the eastern UK coast, we note stronger positive CWL-peak discharge dependence along the north shore of Aberdeen and Wick, gradually weakening towards the south; in a few cases, we find a weak negative association (Fig. 2a–c). The strong dependence on the north shore of Aberdeen could be attributed to the orographically enhanced precipitation owing to the hills on the northern side and cyclones traveling north-eastward to the north of Scotland^24^. The notably weaker correlation across southeast England could be a consequence of persistent drought episodes in this region, followed by failure of groundwater resources to replenish the streamflow during the winter season^59,60^ when high CWL occur. Along the western and southern coast of UK, we find stronger positive dependence in the western part of the south coast, southern Wales and Solway Firth (Fig. 2a–c), which is due to the orographically enhanced precipitation associated with south-westerly airflow in these regions^25^. A rare occurrence of compound flooding along most of the Nordic countries has also been reported in an earlier study^28^.

Apart from analyzing total correlation, we analyzed empirical upper tail dependence for 520 combinations of TG-SG pairs across northwestern Europe, to better understand spatial dependence pattern of extreme events (Fig. 2b,c). The association gets stronger at the upper tail (See Methods). While the values of Kendall’s τ vary from −0.35 to 0.45 (Fig. 2a), the empirical upper tail dependence measures, ${\lambda }_{CFG}$ ranges between −0.24 and 0.52 (Fig. 2b), and ${\lambda }_{LOG}$ varies between −0.24 and 0.59 (Fig. 2c), respectively. The upper tail dependence coefficients (UTDC) show a larger fraction of TG-SG pairs with significant (p-value < 0.05 for 10,000 bootstrap simulations; shown using colored circles in Fig. 2b,c) coefficients than that of the complete correlation metrics. The spatial variation of UTDC shows a distinct pattern with strong positive values of λ_*LOG*_ for Scandinavian countries, whereas the number of sites with significant λ_*CFG*_ values is relatively higher for the region south of 50°N latitude. The differences between the two UTDC estimates are due to the fact that the λ_*CFG*_ estimator approximates the underlying joint distribution function by an extreme value distribution^49^.

The differences in spatial patterns of full versus UTDC dependence could be a consequence of (*i*) a weak correlation between discharge and coastal water levels for the full distributions, where the tide may mask the influence of the surge, and (*ii*) stronger correlations if the tail of the distributions is isolated where the influence of the meteorological component of total water level, i.e. surge, is stronger. Hence, analyzing a wide range of dependence measures between compound flood drivers is crucial for characterizing associated hazards. The density functions of UTDC coefficients (Fig. 2d) show significantly higher values (using the nonparametric Wilcoxon signed-rank test to compare the difference in means of the two samples at 5% significance level) compared to Kendall’s τ, which measures the dependence across the whole spectrum of values. In the case of *λ*_*LOG*_, the density function gets flattened with an elongated right tail, implying strong positive dependence for a number of TG-SG pairs.

Figure S1 presents heat maps of the dependence metrics for tidally influenced and a few selected non-tidally influenced stream gauges (SGs). We find no significant differences in the nature of dependence between tidally influenced and non-tidally influenced SGs (*P*_*Kendall* τ_ = 0.46, *p*_*λCFG*_ = 0.40, and *p*_*λLOG*_ = 0.40, where *p*_*Kendall* τ_, *p*_*λCFG*_ and *p*_*λLOG*_ are the p-values obtained from the nonparametric Kruskal-Wallis test at 5% significance level to assess if significant differences exist between two groups). Figure S1 shows an overall positive dependence as indicated by Kendall’s τ; the stronger dependence at the upper tail is obvious for both types of river gauges. However, in a few cases, we find disparate signs for Kendall’s τ and the UTDC. For example, Kendall’s τ correlations at Dover-Thames and Lowestoft-Thames at Kingston are weakly negative; however, the UTDC metrics show positive dependence. The significant shift of the UTDC distributions relative to the distribution of Kendall’s τ (Fig. 2d) indicates an increased likelihood of compound flooding in those cases, which show relatively weak full dependence or independence.

### Role of extreme coastal water levels in modulating river flood hazards

**Figure 3:**
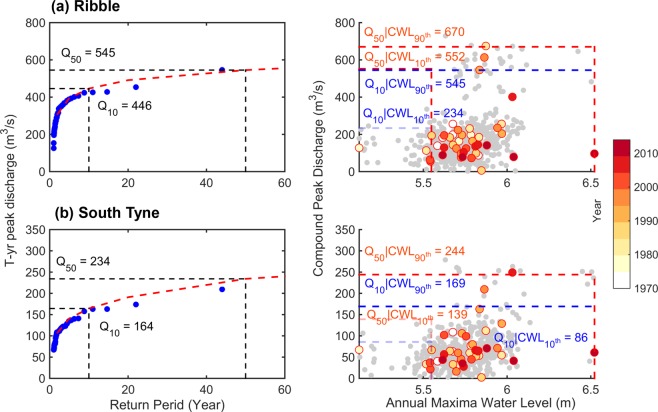
Stronger upper tail dependence relative to complete dependence increases the likelihood of compound flood events: Proof-of-concept illustrations of unconditional (*left panel*) and conditional (on high coastal CWL; *right panel*) flood hazards in UK Rivers along the North shields TG: River Ribble (a, top panel) a tidally influenced river located at a geodesic distance of 157 km and in the River South Tyne (b, bottom panel), non-tidally influenced, located at a geodesic distance of 69 km from the TG. (**a**) Kendall’s τ correlation between Annual maxima CWL and peak discharge for River Ribble is 0.16 with *p-*value = 0.12 [the *p-*value indicates the evidence against the null hypothesis of independence: the smaller (larger) the *p*-value, the stronger is the evidence against (for) the null hypothesis; however, a *p*-value does not indicate the probability that the null hypothesis is true], while empirical upper tail dependence coefficients are ${\lambda }_{CFG}^{Obs}$ = 0.28 (p-value = 0.0054) and ${\lambda }_{LOG}^{Obs}$ = 0.44 (p-value = 0.011). (**b**) Kendall’s τ correlation associated with compound event pairs in River South Tyne is 0.25 with p-value = 0.018, while empirical upper tail dependence coefficients are ${\lambda }_{CFG}^{Obs}$ = 0.35 (p-value = 0.001) and ${\lambda }_{LOG}^{Obs}$ = 0.44 (p-value = 0.013). While circles with shades in yellow and red denote the year of occurrence of the compound event, the one in gray indicates copula-simulated samples. For clarity, return level estimates are rounded to their nearest decimal numbers. Maps are generated using MATLAB 2015a (Version 8.5, http://www.mathworks.com), and then organized and labelled in Adobe Photoshop CS6 Desktop (Version 13.0.1 × 32, http://www.adobe.com) [Software].

As a proof-of-concept, we analyze *T*-year peak discharges of 10- and 50-year events for hypothesized storm episodes in the River Ribble (Fig. 3a; top panel), a tidally influenced river and the River South Tyne (Fig. 3b; bottom panel), a non-tidally influenced river. The unconditional *T*-year peak discharge for the River Ribble at 10- and 50-year events are Q_10_ = 445.9 m^3^/s and Q_50_ = 545.2 m^3^/s, respectively. If we consider compound flooding, the conditional (on the 90th percentile CWL values) *T*-year peak discharge at 10- and 50-year events, are Q_10|90th CWL_ = 544.7 m^3^/s and Q_50|90th CWL_ = 670.2 m^3^/s, respectively (see Methods, ‘|’ indicates conditional on). The severity of both events using the newly developed CHR index is 1.22 and 1.23, respectively. This indicates that accounting for the impact of compound flooding, floods are ~22% more severe than that of the unconditional *T-year* discharge estimates. On the other hand, considering the 10th percentile CWL values, which can be a consequence of a less severe storm, the conditional return level estimates are, Q_10|10th CWL_ = 233.7 m^3^/s and Q_50|10th CWL_ = 552.1 m^3^/s, respectively. Hence, for lower CWL values, the univariate or unconditional return level estimates provide reasonable hazard estimates.

The at-site univariate 10- and 50-year peak discharge for the River South Tyne (Fig. 3b; bottom panel) are Q_10_ = 164.4 m^3^/s and Q_50_ = 234.2 m^3^/s, respectively. The conditional *T*-year peak discharges due to compound flooding are Q_10|90th CWL_ = 169.1 m^3^/s and Q_50|90th CWL_ = 244.1 m^3^/s respectively. This results in the CHR estimates of 1.03 and 1.04 respectively, indicating that compound flood episodes are in the order of 3–4% higher than that of the unconditional flood peak estimates. Considering 10^th^ percentile CWL values leads to Q_10|10th CWL_ = 85.6 m^3^/s and Q_50|10th CWL_ = 139.0 m^3^/s, indicating that univariate measures provide adequate estimates of flood hazards.

In the tidally influenced River Ribble, the flood hazard gets amplified by severe storm-induced high CWL, even if its geodesic distance to the North Shield tide gauge (TG) is larger (157 km) than that of the non-tidally influenced River South Tyne (69 km from the same TG). Further, our results suggest that the analyses based on complete dependence are often inadequate to quantify compound flood hazards that occur at low probability (*e.g*., River Ribble characterized by very low Kendall’s τ correlation, however with significant upper tail dependence, Fig. 3a), since these metrics are more adapted to reflect dependence at the centre of the distribution.

### Assessing the severity of compound flooding: identification of event-specific flood hotspots

Next, we investigate spatial pattern of compound flood severity for three illustrative storm episodes that have caused large insurance losses and were characterized by large spatial spread and high severity^61,62^: Capella [1^st^ to 5^th^ January, 1976], Xynthia [26^th^ February to 7^th^ March, 2010] and Xaver [4^th^–11^th^ December, 2013]. To compare extreme CWL across multiple sites, we calculate the associated CWL anomalies during 1970–2014 (Tables S1–S3). We find that during Capella, ten TGs showed extreme water levels. CWL anomalies of eight out of these ten sites exceeded 1.0 Standard Deviation (SD), with large values exceeding 2.0 SDs at the German and Dutch TGs, Cuxhaven and Den Helder (Table S1). During Xynthia, CWL anomalies exceeded 2.0 SDs in Saint Gildas and La Rochelle along the French coast (Table S2), due to the coincidence of the storm arrival with a high spring tide^63^.

**Figure 4:**
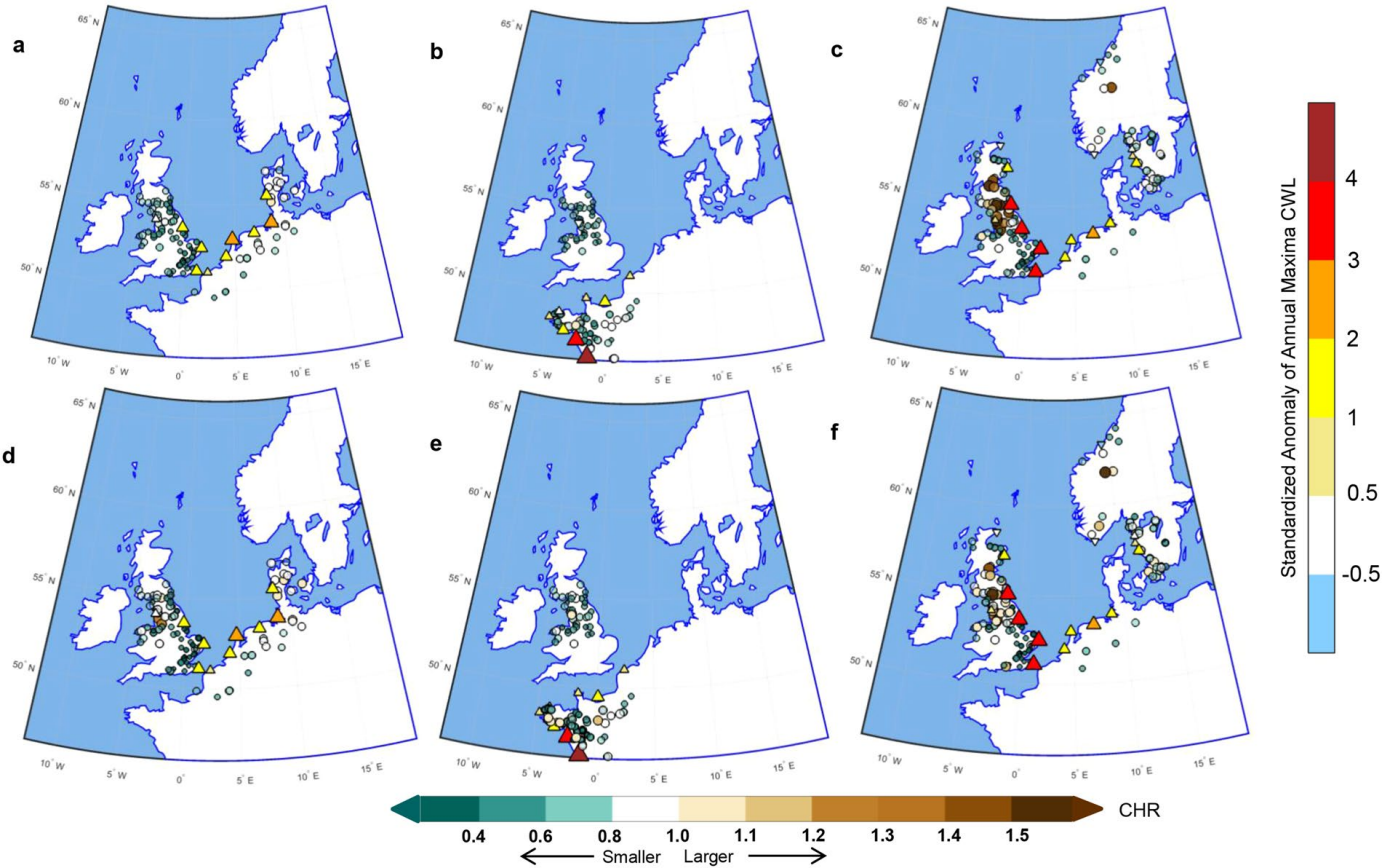
Spatial Variations in compound flood hazards for selected winter storm events. Spatial distribution of CHR index showing compound flooding hotspots for three winter storm episodes: Capella (1^st^–5^th^ January, 1976; **a** and **d**), Xynthia (26^th^ February–7^th^ March, 2010; **b** and **e**), and Xaver (4^th^–11^th^ December, 2013; **c** and **f**) for *T* = 10- (*top panel*) and 50-year (*bottom panel*) return periods. The triangles indicate locations of TG. The colours in the TGs indicate the standardized anomaly of annual maximum CWL, while the size of the triangle is proportional to its value. The upward (positive) and downward (negative) triangles indicate the sign of the standardized anomaly at each TG location. The circles show SG locations where CHR is calculated. The darker shade represents a high value indicating a greater hazard, while a lighter shade denotes low hazard associated with the compound event. Maps are generated using MATLAB 2015a (Version 8.5, http://www.mathworks.com), and then organized and labelled in Adobe Photoshop CS6 Desktop (Version 13.0.1 × 32, http://www.adobe.com) [Software].

Storm Xaver resulted in the highest CWL since 1953 (Table S3) and widespread flooding in UK^64^. In contrast, during Xaver, CWL anomalies over Norwegian coasts remained negative. The greater rate of land uplift along the North Atlantic coast in recent decades as compared to the rate of sea level rise^65^ may have contributed to this effect. The CHR index is used to identify event-specific hotspots. Figure 4 shows the CHR values for the return periods, *T* = 10 (Fig. 4a–c) and 50-years (Fig. 4d–f) for the three selected storms. As an indicator of compound flood hazard, we estimated CHR for flood events with 10- and 50-year return periods. The CHR for the 10-year event indicates moderately severe discharge, whereas CHR at higher return level, i.e., 50-year event, denotes severe discharge and the related maps can be used to assess flood exposure and risk of population and assets. At *T* = 10-year, at least one of the SGs has CHR larger than 1. For storm Capella (Fig. 4a,d), most of the SGs along the coasts of the Netherlands, Germany and Denmark show CHR values close to 1, which indicates that the severity of compound flooding is comparable to the unconditional, at-site *T-*year peak discharge estimates. CHR values smaller than 1, among UK river basins, indicate that the severity of compound flooding was smaller than that of the *T-year* fluvial peak discharge.

We show spatial maps of CHR at 50-year return period to identify the hotspots associated with synoptic meteorological conditions that have caused compound flooding. For storm Xynthia, (Fig. 4b,e), a large number of SGs on the French coast show CHR value of either close to or larger than 1, owing to the exceptionally high CWL (Table S2) caused by the co-occurrence of associated storm surge and high spring tide during this event^63^. The largest value of CHR = 1.12 was reported at River Orne along Le Havre coast (the geodesic distance of 147.5 km), which is tidally influenced. The CWL at Le Havre exceeds 1-SD level during storm Xynthia (Table S2). Likewise, we find a CHR value of 1.08 for the non-tidally influenced River Dronne (at a geodesic distance of 168.5 km) along the coast of La Rochelle; the CWL anomaly of which exceeds 4.0 SD (Table S2). For storm Xaver (Fig. 4c,f), the spatial pattern of CHR shows more widespread flooding as compared to the other two storms. The two of the SGs along the coast of Wales show CHR values of more than 1.2, among which one of the SGs is tidally influenced.

**Figure 5:**
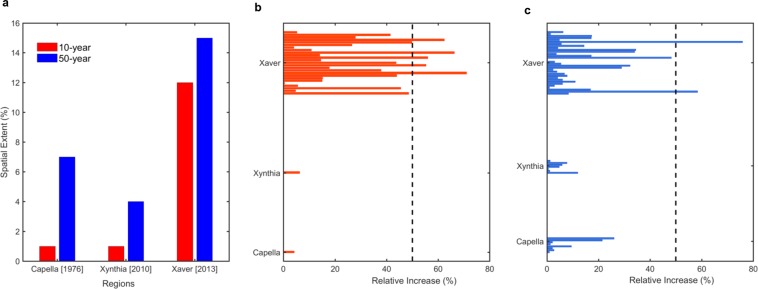
The fraction of TG-SG pairs showing an increase in the likelihood of compound flood hazards for the three winter storm episodes (**a**) Fraction (expressed as a percentage) of TG-SG pair with an increase in *T-year* peak discharge associated with compound event relative to at-site peak discharge. Percentage relative increase in *T-*year peak discharge for (**b**) 10- and (**c**) 50-year events. The increase in discharge is quantified as the relative difference between the magnitude of the *T-*year flood peak conditional on AMWL and the seasonal maxima (November-March) at-site *T-*year peak discharge expressed as a percentage. The horizontal bars in red (figure b) and blue (Figure c) show TG-SG pairs with an increase in flood hazard. The dotted vertical line (in black) indicates the relative increase of the order of 50%. Figures are generated using MATLAB 2015a (Version 8.5, http://www.mathworks.com), and then organized and labelled in Adobe Photoshop CS6 Desktop (Version 13.0.1 × 32, http://www.adobe.com) [Software].

Figure 5 shows the increased likelihood of river floods associated with each of the winter storm episodes. The largest spatial coverage is associated with 2013’s storm Xaver (Fig. 5a), which was characterized by severe storminess across a wide area^61^, resulting in a number of TGs to exceed the 2-SD anomaly level, especially along the coast of UK (Table S3). We find a large number of stations exceeding CHR values of more than 1 along the coasts of UK, Sweden and Norway. At return period *T* = 10-year (Fig. 5b), the relative increase in peak discharge ranges between 0.4 and 71% with five gauges showing more than 50% rise, out of which three gauges are tidally influenced. The largest increase (71%) is noted for the TG-SG pair ‘North Shields – Carron’, a non-tidally influenced river at Firth of Forth, Scotland, which exhibits a relatively strong positive dependence significant at 5% level (Kendall’s τ = 0.24 with *p*-value = 0.022; ${\lambda }_{CFG}$ = 0.33, *p-*value = 0.003 and ${\lambda }_{LOG}$ = 0.52, *p-*value = 0.008). In contrast to most other catchments in the eastern UK, this strong dependence in the area to the north of Firth of Forth is a consequence of prevalent cyclone storm tracks resulting in orographically enhanced precipitation and high streamflows^24^.

At return period *T* = 50-year (Fig. 5c), the relative increase in return level for the compound flooding event is more than 70% for tidally influenced River Eden (nearest TG North Shields at a geodesic distance 97.27 km) in the UK, which shows a very weak (insignificant) positive complete correlation (Kendall’s τ = 0.062, p-value = 0.56), however, significant upper tail dependence (*λ*_*CFG*_ = 0.21, p-value = 0.06 and *λ*_*LOG*_ = 0.42, p-value = 0.015). Interestingly, the standardized CWL anomaly at North Shields exceeds 3.0-SD during the 2013 storm (Table S3) with the largest number of SGs show an increase in *T-year* peak discharge around the TG. A ~ 58% increase in 50-year peak discharge is observed for the non-tidally influenced River Otta near TG Heimsjoe along the Norwegian coast. For this TG-SG pair, the Kendall’s τ correlation is 0.065 (p-value = 0.065) whereas a significant empirical UTDC is observed for the CFG case, ${\lambda }_{CFG}$ = 0.184 (p-value = 0.032). While the standardized annual maxima water level anomaly at Heimsjoe during storm Xaver (on 5^th^ December 2013) was slightly negative (Table S3), the increase in the likelihood of compound floods along the Norwegian coast clearly demonstrates, how successive climate extremes could produce an extreme impact even when either extreme in isolation would not be considered as particularly severe^63^. In addition, we find that 24–36% tidally influenced SGs and 10–12% non-tidally influenced SGs show increased fluvial flood hazard. Taken together, our analysis suggests the following: *(i)* Increased fluvial flood hazard is associated with high CWL resulting from a severe storm episode. *(ii)* A stronger dependence between extreme CWL and river flow, especially at the upper tail of the distribution, amplifies the compound flood hazard.

## Discussion

Compound flooding, *i.e*., the simultaneous or successive occurrence of high coastal and river water levels, results from clustered or multi-variable drivers that may produce extreme impacts even when impacts from either driver in isolation would not be particularly severe^6,66–68^. The understanding of such events thus is of high interest. Most of the TGs along the northwestern European coastline have experienced an increase in relative sea level^9,69,70^. As global warming amplifies extreme sea levels over the next decades^10,71,72^, flooding from high tides is expected to occur more frequently causing disruption to LECZ regions. To the best of our knowledge, this paper is the first to quantify the severity of riverine flooding by developing a dimensionless index, representing extreme CWL – peak discharge co-variability. We demonstrate the applicability of this index using three catastrophic storm episodes. Further, we find that the extremal dependence is robust for 36 (${\lambda }_{LOG}$) to 60% (${\lambda }_{CFG}$) of TG-SG pairs considering upper tail dependence metrics, while the complete correlation between extreme CWL and peak discharge is significant only for 20% of TG-SG combinations. While most of the earlier assessmants^4,26,36,56^ have used copula-based simulation to analyse the joint frequency of compound floods (either CWL or surges and discharge), very few of them have assessed the severity of conditional peak discharge considering the role of the extreme CWL as the contributing driver of flood. Other novelties include the consideration of the catchment response in determining the lag time of fluvial peak discharge and the selection of independent and identically distributed events, an aspect not thoroughly considered earlier^3,6,19,50^. By leveraging *in situ* observations, our study avoids some of the uncertainties associated with satellite measurements^73^ and numerical model chains^19,50,55,57^.

Some of the caveats of the study include: we assume that the response time of a river is a function of catchment size only, which is based on an analytical derivation of both dynamical and statistical properties of a watershed^52,53^. The response time may be affected by the presence of dams or other anthropogenic influences, which may not be adequately reflected by the empirical equation. Finally, the period analysed is limited to 45 years (1970–2014) to cover large parts of the northwestern European coastline based on the best quality available records. Given the limited record length, we assume that the effects of changes in the time series are not large.

Globally, Europe ranked third next to Asia and North America in terms of exposed population and assets to extreme CWL; among the European cities situated at the LECZ, two of the cities are located in northwestern Europe with exposed assets over 240 $ Billion, estimated in the year 2007^74^. Even if protection standards of the cities across northwestern European coasts are high^12^, distributions of exposed population and assets to compound flooding across LECZ are likely to translate into unprecedented disasters^58,75–77^. The proposed CHR index allows the quantitative evaluation of the relative roles of extreme sea levels in modulating inland flood hazard. The dependence between high CWL and peak discharge fattens for extreme events; ignoring this linkage could lead to underestimation of concomitant flood hazard^49,78^. Our main finding – high CWL and stronger storms elevate fluvial flood hazard – is critical along the densely populated northwestern European LECZ, when the increasing risk of fluvial floods^12^ coincides with the risk of coastal flooding^10,79^ in a changing climate.

The proposed approach could be used in multi-fold ways. Maybe most importantly, it allows to understand the dependence between high CWL and high river discharge and to quantify the probability in river flooding given extreme CWL. The derived index could add value to flood loss assessments by communicating the results not only to populations residing on coasts but also to those living in inland areas in order to better prepare financially to ensure resiliency to compound hazards. The probabilistic framework can be extended to include additional flood drivers, such as co-variability of major large-scale atmospheric circulation patterns. A potential candidate is the North Atlantic Oscillation (NAO) that influences flow regimes in northwestern Europe especially during winter season^80^. The causal links established here are further convoluted by regional and global changes, including direct and indirect human interventions^81,82^, which we plan to explore in a future assessment.

## Datasets and Methods

### Coastal water level data

Hourly sea level observations (in meter) from 1970 to 2014 for northwestern Europe (approximately 46°–66°N and −12.5°W~19°E) were obtained from 32 tide gauges (TG) archived at Global Extreme Sea Level Analysis version II database^83^. Annual maximum water levels (AMWL) were extracted from the hourly time series as an indicator of extreme CWL. To compare AMWL values across space and time, standardized anomalies of AMWL time series were calculated, computed as the magnitude of AMWL anomaly (i.e., departure from its long-term mean) divided by the standard deviation (SD). We consider “moderate” water levels as those that remain within ± 1 SD, “severe” as those that are above + 1 SD and below 1.5 SD, and “extreme” as those that exceed 1.5 SD [Tables S1–S3]^84^.

Each TG contains more than 40 years of high-quality relative sea-level records since the 1970s. The database has been extensively used in extreme sea level analysis^85^. Relative sea-level data at Hoek van Holland TG was obtained from Directorate for Public Works and Water Management, Rijkswaterstaat, the Netherlands. Most of the TGs contain records of hourly temporal resolution. A few TGs have higher sampling frequencies, such as TGs from the UK and Norway with sampling frequencies of 15 and 10 minutes from 1993 and 2001 onwards, respectively. For consistency, observations of higher sampling frequencies were averaged to hourly resolution by calculating the median of the *n* values within each hour^85^.

### Fluvial discharge data and selection of streamflow gauges

Daily river discharge data from 241 stream gauges (SG) are obtained from the archived hydrometric observations from Global Runoff Data Centre (GRDC)^86^. Following previous studies^26,36^, we select the SGs that are within *s*′ = 200 km radius around the TGs. The choice of *s*′ was based on an earlier study^11^ that suggests tidal motion may propagate as much as and often more than 200 km inland freshwater systems. Further, we obtain a catalogue of SGs that shows tidal bore^11^ characteristics around the European coast from the USGS Technical report^87^. Based on the available literature^87–90^, we classified the selected SGs into “tidally influenced” and “non-tidally influenced” categories. Figure S2 shows the spatial distribution of TGs and SGs (both tidally influenced and non-tidally influenced) considered in this study. It should be noted that our approach does not only consider tidally reversing currents as possible cause of compound events, but also the fact that compound events may result from the co-occurrence of high coastal water level and river discharge that stem from common meteorological drivers owing to a severe storm episode. Hence, even though a specific TG may not be physically connected to a SG through a river outlet, a causal connection may exist in a meteorological sense based on the strength of dependence between them^91^. Figure S3 demonstrates that positive dependence exists between TGs and SGs across large distances.

### Identification of compound events

Assessing the severity of compound extremes is a challenging task^66^. To establish pairs of TG–SG extreme event time series, we use the AMWL values observed at the TGs as a starting point. For each TG–SG pair, we shift the SG time series by a lag time for which we use the watershed response time of the specific SG. We then search for the highest peak in the shifted SG time series within an interval of ± 7 days from the day of occurrence of the AMWL event.

The adoption of a lag time is motivated by the consideration that, if high CWL and river discharge show some dependence, this dependence should stem from a common meteorological cause^3^. While high CWL due to storm surges are frequently associated with synoptic low pressure systems and onshore winds, a moisture-laden air mass in the catchment leads to extreme precipitation causing riverine flooding. Whereas the effects of such systems are seen directly at the coast, the precipitation they bring to the river catchments needs to propagate through the watershed to be felt at downstream gauges. Hence, we use the watershed response time *d,* which is largely related to catchment area, A_D_ and is given as^52–54^

1 $$d=2.51\,{A}_{d}^{0.4}[hrs]=0.11\,{A}_{d}^{0.4}[days]$$

where A_D_ is in km^2^. Eq. [1] is derived by assuming the shape of the drainage basin is semicircular, in which the flow distance is proportional to ${A}_{D}^{0.5}$. As *A*_*D*_ increases, the discharge and size of the channel will increase, resulting in an increase in the hydraulic radius and a decrease in the relative roughness. This will, in turn, reduce the effect of *A*_*D*_ on response time, leading to^52^ *d* ∝ ${A}_{d}^{0.4}$. Figure S4 presents the spatial distribution of catchment response time over northwestern Europe. The hydrologic response time varies between 1 and 13 days depending on the catchment area at each of the river basin. About 50% (120 out of 241) of SGs show *d* value of 1-day. The largest response time is observed for the River Rhine (catchment area = 1, 60, 800 km^2^) at Lobith, the Netherlands with a *d* value of 13-day, followed by the River Elbe (catchment area = 1, 31, 950 km^2^) at Neu-Darchau, Germany with a *d* value of 12-day. A sensitivity test (Figure S5) using different lag-times (including the one obtained from Eq. 1) reveals that the dependence is not sensitive to changes in the time lag, as the variation of the coefficients around the selected time lags is negligible.

### Identification of storm events

We identified three independent historical storms since the 1970s, that caused large insurance losses, and that were characterized by relatively large spatial extent and extremely severe storm episodes as indicated by meteorological indices (such as wind speed and storm size), archived at Extreme Wind Storms (XWS) catalogue for Europe^61^. This catalogue includes all major storms that occurred between 1979 and 2013, the period covered by ERA-Interim. Prior to 1979, we include storm Capella (January 1976) with reported original losses in the order of ~ €1bn and the highest CWL observations in many of the TGs^62^.

### Dependence between AMWL and peak discharge

While the complete dependence between 520 TG – SG pairs is analysed using Kendall’s tau (*τ*), the Upper Tail Dependence Coefficients (UTDC; *λ*) are determined using the Capéraá-Fougéres-Genest estimator (*λ*_*CFG*_) and LOGarithm of the diagonal section of the Copula (*λ*_*LOG*_). The metric *τ* measures the strength of a monotonic dependence between two non-normally distributed random variables based on concordant and discordant pairs. We preferred Kendall’s *τ* over Spearman’s ρ since the former offers better estimates of population parameter with smaller asymptotic variance; it is, hence, less susceptible to outliers^92^. Kendall’s *τ* ranges between −1 and +1, and the positive (negative) value of *τ* indicates perfect association (disagreement) between variables. However, correlation only indicates the degree of association between two variables and does not capture the dependencies well, especially at the tail (i.e., events with low probabilities), because it is based on the full range of the data. To analyse correlation at the tails of the bivariate extremes, we use two empirical UTDC metrics that infer concordance on the upper quadrant tails. The statistical significance of the UTDCs is established by drawing *N* = 10,000 random bootstrap samples and then calculating the p-value of the test (i.e., probability of observing a stronger correlation by chance) from the simulated bootstrapped samples using standard percentile-based approach. We report statistical significance at 5% significance level, unless otherwise specified.

### Multivariate statistical analysis

In general, risk is estimated as^93^: Risk = probability (of flooding) × consequence (of flooding), where the term probability (or likelihood) refers to the probability of extreme events with a potentially significant impact. Here we focus on the probability part of the equation.

The marginal distributions of compound flood drivers are modelled using a suite of distributions based on the literature (Supplementary Statistical Methods A.1). While Table S4 presents marginal distribution fits of the selected TG-SG pairs, Figs S6–S7 compare the Probability Density Functions (PDFs) and the Cumulative Density Functions (CDFs) of the corresponding pairs with respect to the empirical distribution as estimated using Gringorten’s plotting position formula^94^. The marginal distribution fits (Figs S6–S7) of the individual flood drivers suggest a satisfactory fit between theoretical and empirical distributions. To model nonlinear associations between AMWL and river peak discharge, we use four different families of copulas (Table S5), widely applied in hydrology^26,29,95^: Clayton, Gumbel-Hougaard, Frank and Student’s *t*. Two of them, Gumbel-Hougaard and Student’s *t*, show upper tail dependence. The Archimedean class of copula families, Clayton and Gumbel-Hougaard, can only model positive dependence while the remaining two families, i.e., Frank and Student’s *t*, can model a wide range of dependence including a negative Kendall’s τ. It should be noted that we model the copula-based joint distribution when the maximum of the two empirical UTDC metrics shows a positive value ($\max ({\lambda }_{U}^{CFG},{\lambda }_{U}^{LOG}) > 0$) [Fig. S3], even when the total correlation values are weakly negative. In this way, we retain only those pairs for which the maximum of the two UTDC estimates is positive. This considerably reduces the computational effort for the analysis, as this allows us to include only those pairs whose tails show positive dependence and exclude around 10% pairs (55 out of 520 TG-SG pairs) with negative upper tail dependence. More importantly, it allows to identify pairs with complex dependence behaviour, i.e. overall negative dependence but positive tail dependence. Relying only on the overall dependence using Kendall’s correlation would underestimate the occurrence of compound floods. The goodness of fit of such pairs is assessed using Frank and Student’s *t* copula families, respectively.

We estimate the parameters of the copula models using the maximum pseudo-likelihood method^96^ (Table S5). First, we assess the suitability of the hypothesized copula family qualitatively by visually inspecting^95^ superimposed scatter plots of observed versus 1000 randomly generated synthetic data from the copula (Figure S8) to assess the adequacy of the selected copula families to model bivariate dependence. Further, we assess the goodness of fit (GoF) of the copula models^97^ using the Cramér-von Mises distance (*S*_*n*_), i.e. the integrated squared difference between empirical and parametric copula distributions. We then evaluate the statistical significance of the test through *p*-values obtained via parametric bootstrap for *S*_*n*_ at *n* = 500 replications (Table S5), indicating that our findings are robust to the choice of copulas. We further evaluate the adequacy of the selected model in capturing the upper tail dependence using Mean Error to Standard Error (MESE) statistics (Supplementary Statistical Methods A.2)^98^. The results of upper tail dependence coefficient tests for the selected copula families are listed in Table S6. Figure S9(a) shows the location of SGs with the best selected copula families for each of the TG-SG pair. Figure S9(b) compares the MESE statistics of tidally influenced versus non-tidally influenced SGs. Figure S9(b) indicates the MESE variability associated with *λ*_*LOG*_ coefficients are larger than that of the *λ*_*CFG*_ coefficients. The overall GoF test suggests that 56% (262 out of 465) of TG-SG pairs are satisfactorily modelled by Gumbel-Hougaard and Student’s *t* copula families.

### Compound hazard ratio (CHR) and identification of hotspots

We propose a dimensionless multivariate index, Compound Hazard Ratio (CHR), which is defined as the ratio of the conditional *T*-year peak discharge assuming AMWL as the covariate and the unconditional *T*-year seasonal maxima (November-March) fluvial peak discharge.

The index is motivated by the Flood Ratio approach^99^ for the assessment of inland flooding associated with predecessor rain events. While previous assessments of compound flood hazards were limited to moderately severe events, i.e., *T* = 10- or 25-year return periods^4,28,36,99^ and relied on a particular family of distributions, such as Generalized Extreme Value (GEV)^99^, we show spatial variations of the compound flood severity using the newly developed CHR index for both moderately severe (*T* = 10-year) and severe (*T = *50-year) events. The index is derived from the copula-based conditional *T*-year return period (i.e., the severity of the event expected to occur, on an average, once in every *T* years); hence, it offers flexibility for the choice of the marginal distributions.

The *CHR* is expressed as:

2 $$CHR=\frac{{Q^{\prime} }_{T}}{{Q}_{T}}=\frac{{C}_{Q|CWL=cwl}^{-1}[1-\frac{1}{{T}_{Q|CWL}(q|cwl)}]}{{F}_{Q}^{-1}[1-\frac{1}{{T}_{Q}(q)}]}$$

where *Q*′_*T*_ denotes the conditional *T*-year peak discharge given AMWL, estimated using copula-based conditional distribution, *Q*_*T*_ indicates the at-site unconditional *T*-year peak discharge and CWL denotes coastal water level at TGs. ${C}_{Q|CWL=cwl}^{-1}$ and ${F}_{Q}^{-1}$ denote inverse quantile transformation of copula-based and marginal distributions. While the at-site *T*-year return period of peak discharge, *T*_*Q*_(*q*) is given by ${T}_{Q}(q)=\frac{1}{1-{F}_{Q}(q)}$, the conditional return period, ${T}_{Q|CWL}(q|cwl)$ of peak discharge (the vertical bar “|” means conditional on) at coastal water level, CWL = *cwl* is given by

3 $${T}_{Q|CWL}(q|cwl)=\frac{1}{1-{C}_{Q|CWL=cwl}}$$

where *F*_*Q*_(*q*) denotes the distribution of peak discharge assuming independence to CWL, ${C}_{Q|CWL=cwl}$ indicates the copula-based conditional distribution of the bivariate pair *Q* (peak discharge) - *CWL* (high coastal water level) for certain values of *q* and *cwl*, representing the quantiles of *Q* and *CWL*, respectively. We derive the copula-based conditional distribution of peak discharge at each SG locations for a given AMWL value. The AMWL value derived from total coastal water level represents a stochastic process^4,45^ that composed of all three elements, *i.e*., astronomical tides, mean sea level and non-tidal residuals, and holds a dependence pattern with river discharge. Further, our definition of compound floods includes conditional expectations of river floods on extreme CWL of both tidally influenced river (indicated by Mechanism 1) and non-tidally influenced river (indicated by Mechanism 2). Hence, we include the total water level in the analysis. We estimate the design peak discharge by back-transforming it to the original unit using the marginal distribution of flood peak at each station location. From the historical compound event time series, we extract the AMWL and corresponding peak discharge for the selected storm episode. Using these two values, we compute unconditional and conditional return levels, and the CHR, of that particular storm event. In northern-western Europe, the storm season (November-March) is often characterized by high river flows^100^. Hence, we consider seasonal maxima (November-March) of daily streamflow records for the at-site frequency analysis. The CHR = 1 indicates a perfect agreement between conditional *T*-year peak discharge and local *T*-year fluvial flood discharge. CHR values larger (smaller) than 1 indicate hazards of compound flooding is larger (smaller) than that of the seasonal at-site *T-*year peak discharge.

We define compound flooding hotspots as the locations with a positive upper tail dependence. This definition includes TG-SG pairs with positive or weakly negative overall correlation coefficients. Although the overall dependence measure Kendall’s τ is based on the ranks of the observations that measures the extent of concordance or discordance, it does not attribute sufficient weight to the extreme values when the focus is on the tails of the distributions^78^. Since our goal is to identify hotspots for compound extremes with 10% (i.e., 10-year) and 2% (i.e., 50-year) exceedance probability, the upper tail dependence is a more suitable measure than the overall rank correlation coefficient for the copula-based dependence modelling.

## Supplementary information

Supplementary Information
