# Supplementary material for: Extreme Coastal Water Levels Exacerbate Fluvial Flood Hazards in Northwestern Europe
Source: Sci Rep. 2019 Sep 11;9:13165. doi: 10.1038/s41598-019-49822-6 (PMC6739477; doi:10.1038/s41598-019-49822-6)
Supplement: Supplementary file 1 — Supplementary Information [file 41598_2019_49822_MOESM1_ESM.pdf]

Supplementary Information
for

Extreme Coastal Water Levels Exacerbate Fluvial Flood Hazards in Northwestern Europe

Poulomi Ganguli[1,2*], Bruno Merz[2,3]

[1]Agricultural and Food Engineering Department, Indian Institute of Technology, Kharagpur, India
[2] German Research Centre for Geosciences (GFZ), Potsdam, Germany
[3] Institute of Earth and Environmental Sciences, University of Potsdam, Potsdam, Germany

E-mail: pganguli@agfe.iitkgp.ac.in

A. Supplementary Statistical Methods

A.1 Marginal Distributions of Compound Flood Driver

Based on prior literature[1–3], the marginal distributions of AMWL and peak discharge are modeled using a suite of four distributions: three–parameter Generalized Extreme Value (GEV), two–parameter Gamma, two–parameter Lognormal and the non-parametric Kernel density estimator with kernel type – Normal kernel function. While we estimate parameters of the parametric distributions via the maximum likelihood estimator, the optimal bandwidth (h_{opt}) of the kernel function is chosen using the equation, $h_{opt} = \left(4/3n\right)^{0.2}\sigma$ where σ is the standard deviation and n is the length of the time series. We check the performance of the marginal distribution fits using the distance-based Akaike Information Criterion corrected for small sample size (AICc)[4] between theoretical and rank-based empirical distributions[5]. Since we are interested in modelling extremes, following previous studies[5,6], we estimate empirical distribution using rank-based Gringorten's plotting position formula.

A.2 Upper Tail Dependence Test for the Best Selected Copula Family

Once we find the best copula family using the GoF test, we check the performance of the selected copula family in simulating upper tail dependence coefficients (UTDC). For comparing the performance of selected copula families in simulating UTDC statistics, we calculate $\hat{\lambda}_U^{CFG}$ using simulated random samples of size n (*i.e.*, same as the size of the observed sample) from the copula. The computation is repeated over I = 500 bootstrap runs and the corresponding mean $\mu\left(\lambda_U^{Sim}\right)$ and standard deviation

$\sigma\left(\lambda_U^{Sim}\right)$ values are calculated for each of the UTDC estimate. Finally, we compare the empirical versus copula-based UTDC estimates using MESE statistics.

For this, we generate random samples of size *n* (where *n* is the size of the observed sample) from the selected copula family for *I* = 500 bootstrap runs. MESE quantifies the sample bias normalized by the sample standard error. The sample bias[7] is computed as the absolute difference between the sample mean $\hat{\mu}\left(\hat{\lambda}_n\right)$ from the copula and the empirical estimate of λ, whereas the sample standard error is the sample standard deviation $\hat{\sigma}\left(\hat{\lambda}_n\right)$. The sample mean $\hat{\mu}\left(\hat{\lambda}_n\right)$ and sample standard deviation $\hat{\sigma}\left(\hat{\lambda}_n\right)$ are the estimates of $\lambda_{n,i}$, where i = 1, …, 500 depending on the sample size *n*.

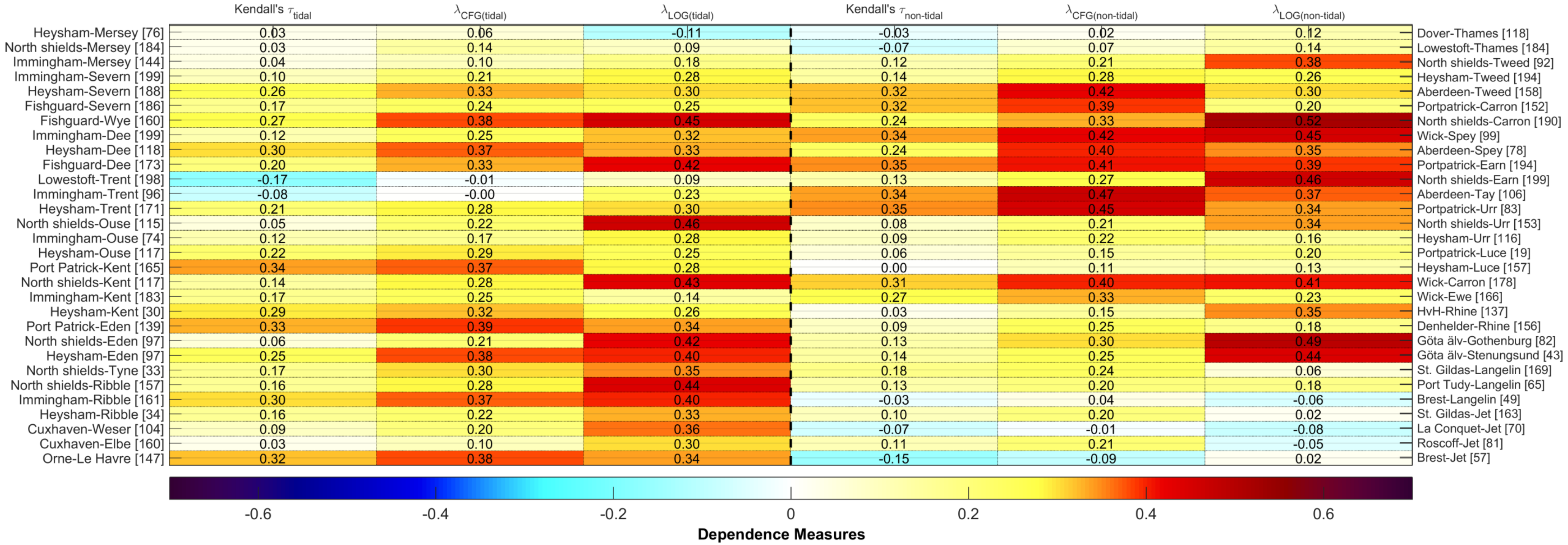

Figure S1. Dependence between AMWL and peak discharge for tidally influenced (left column) and selected non-tidally influenced (right column) SGs. The dependence metrics Kendall's τ indicates complete dependence while λ_{CFG} and λ_{LOG} denote empirical upper tail dependence. The geodesic distance (in km) from the coast for each TG-SG pair is listed in brackets.

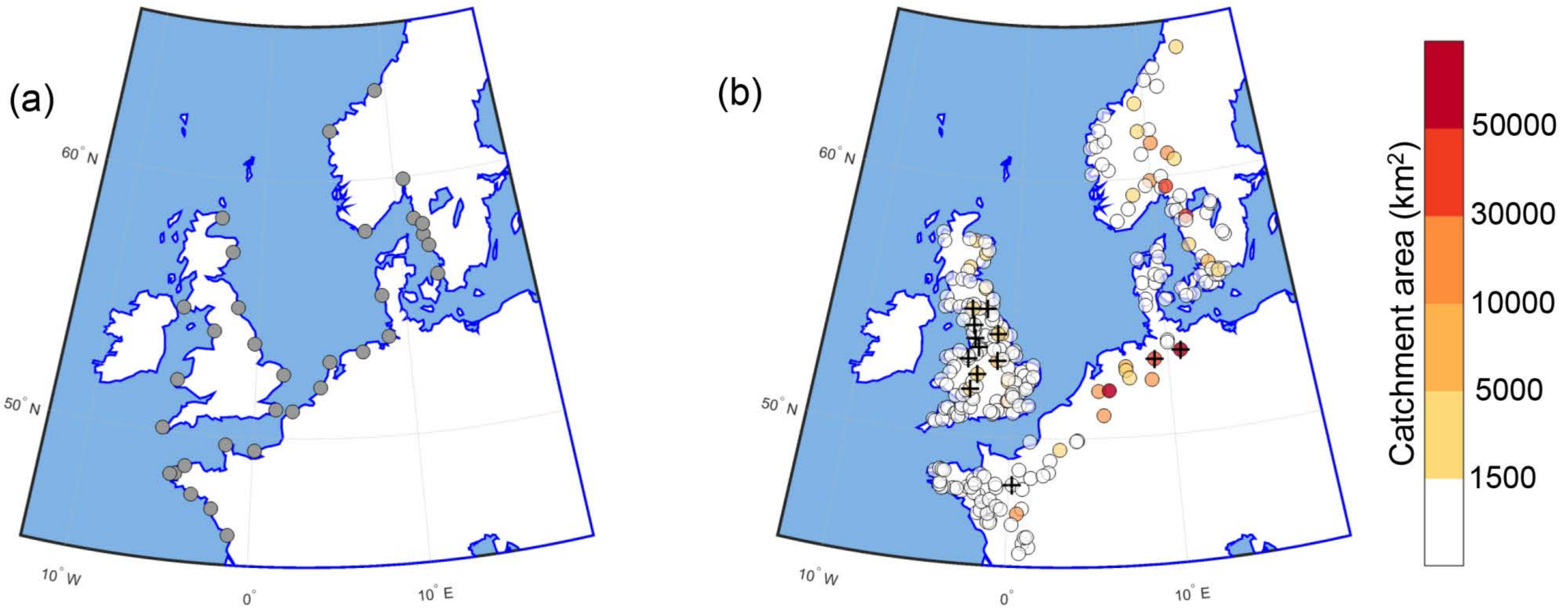

Figure S2. Spatial distributions of TGs and SG locations. (a) The location of TGs along the European coast is shown using circles with grey colour. (b) The circles indicate locations of SGs with shades in the circle indicate the size of the catchment; darker hues show the larger catchments, whereas the lighter shades indicate the smaller catchments. The locations of tidally influenced SGs are marked with a 'plus' sign.

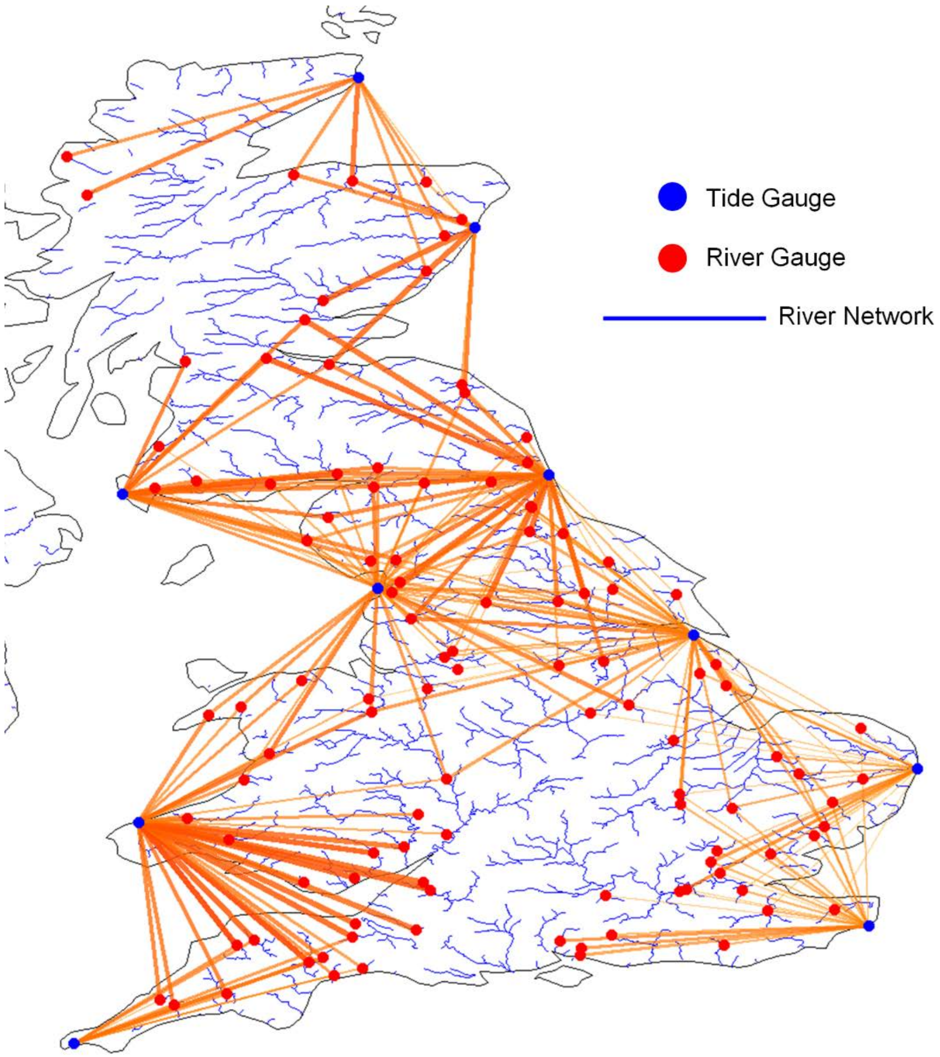

Figure S3. Pairing of TG and SG for a representative region in northwestern Europe. The spatial connectivity is based on the strength of dependence. We consider only those pairs in which the maximum of the two UTDC estimates is positive. The darker and thicker lines represent stronger links, whereas thinner and lighter lines show weaker links between the pairs.

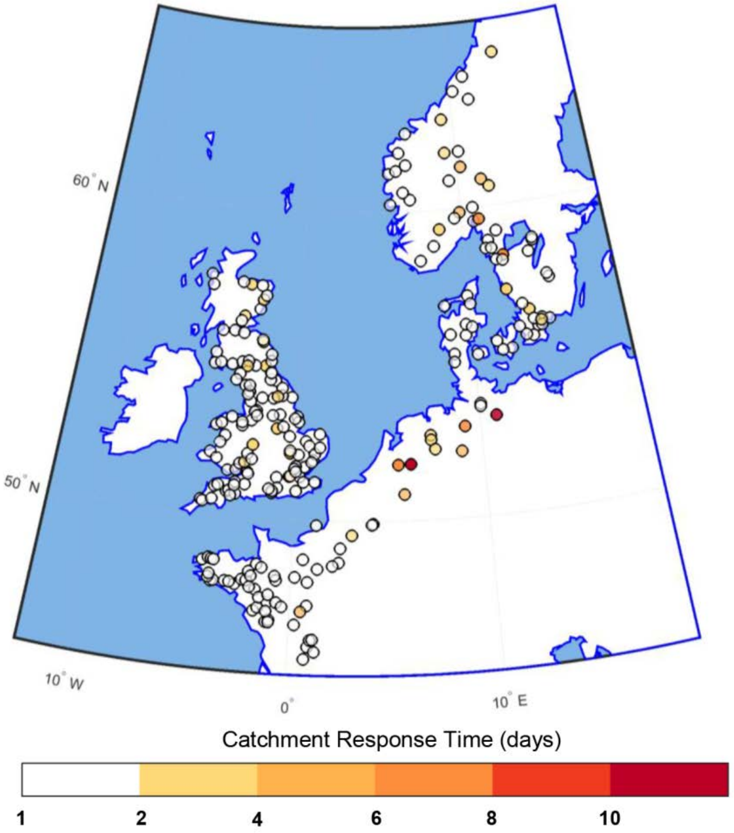

Figure S4. Spatial distribution of watershed response time (in days) as a function of catchment area.

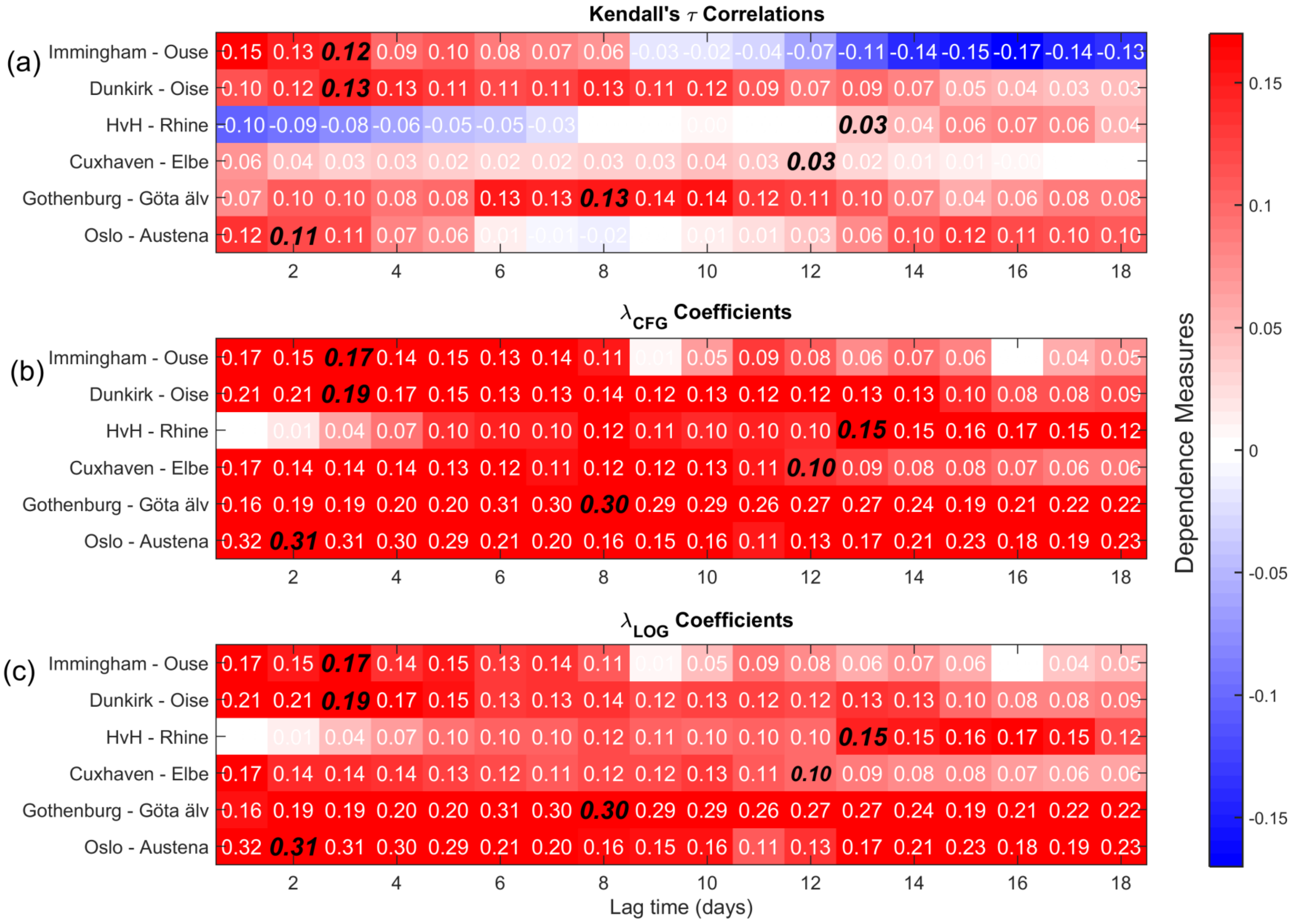

Figure S5. Dependence patterns of extreme CWL versus *d-day* lagged river discharge within ±7 days of occurrence of the extreme event. The dependence value with selected time lags is shown in black using bold italic fonts, where the lag-time is calculated assuming a nonlinear relationship between response time and catchment area.

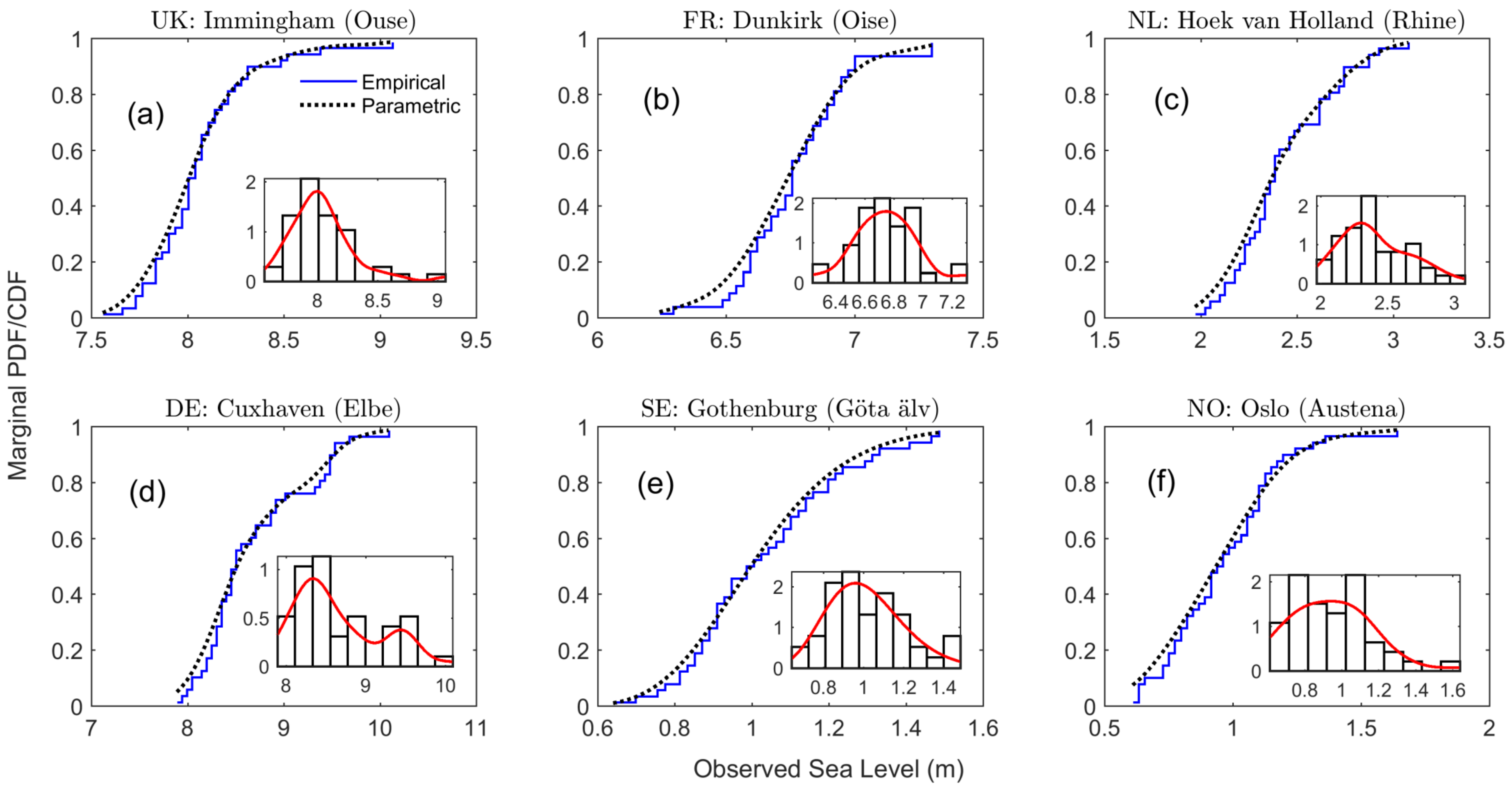

Figure S6. Marginal distribution fit of compound events. Marginal distribution fit of AMWL in the selected tide gauge (TG) – stream gauge (SG) pairs; corresponding SG name is shown in brackets.

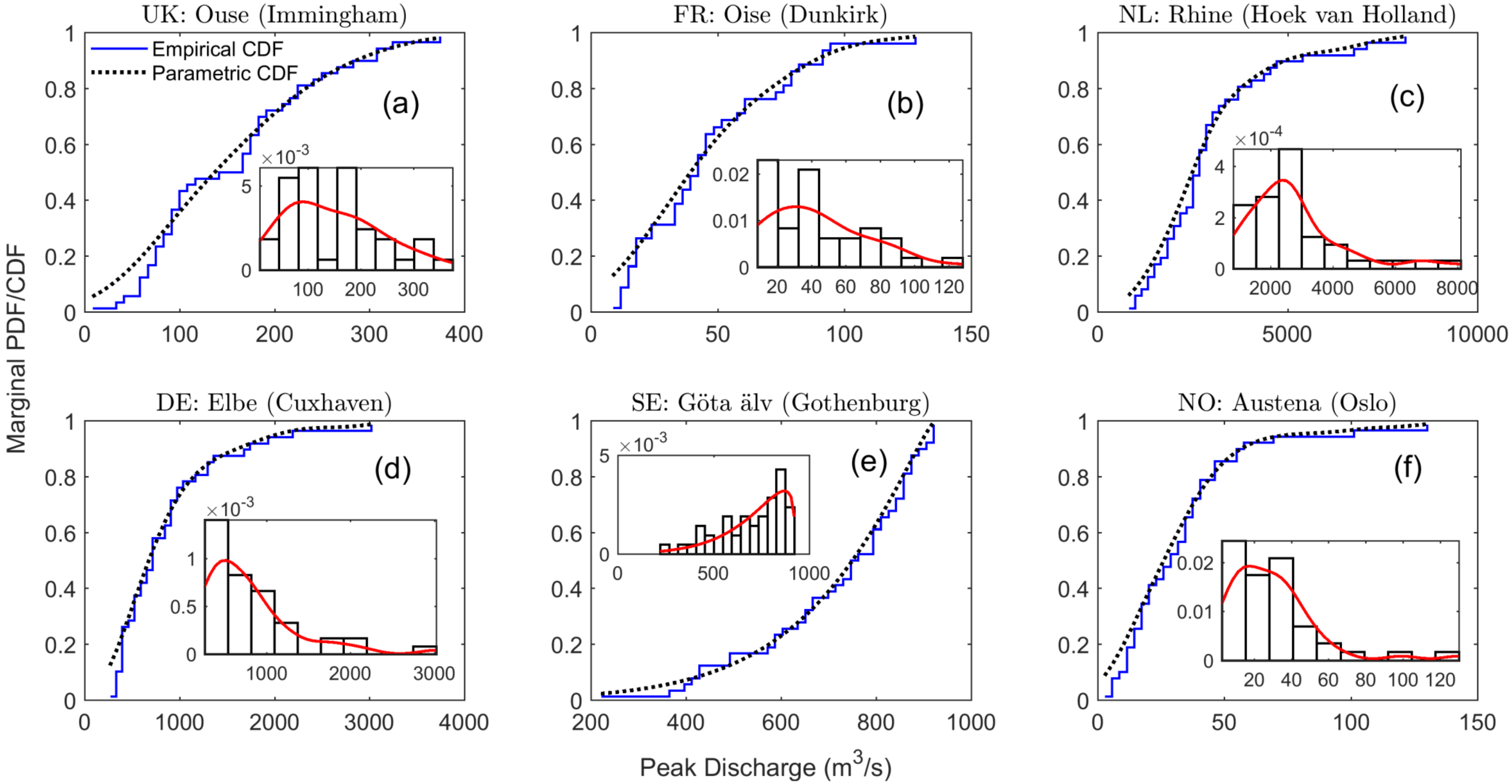

Figure S7. Marginal distribution fit of compound events. Marginal distribution fit of peak discharge in selected TG-SG pairs; corresponding TG name is shown in brackets.

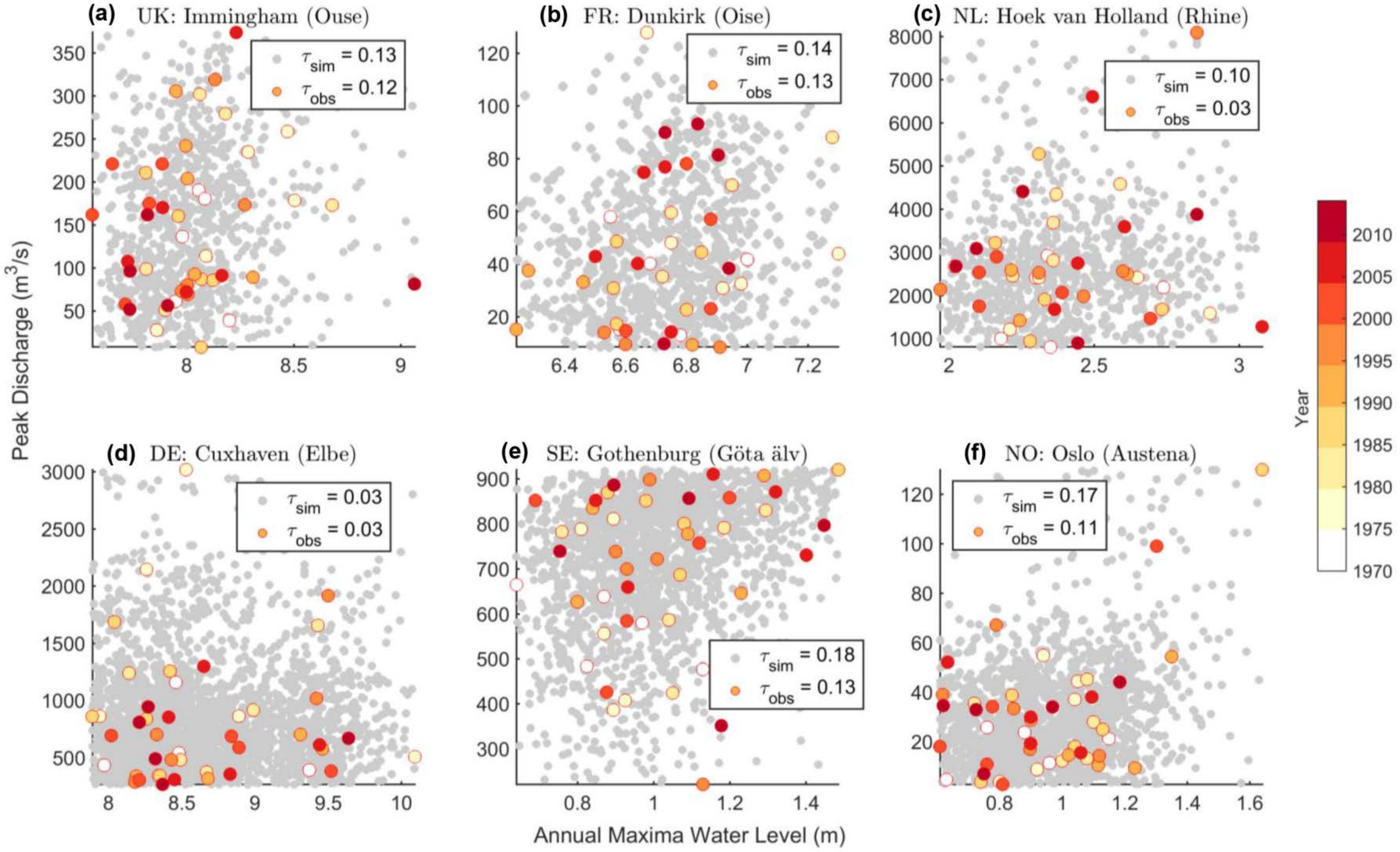

Figure S8. Simulation of compound events. Scatter plots of AMWL and lagged daily peak discharge for selected TG-SG pairs during 1970 – 2014 (in shades ranging from white to red). Simulated samples (*n* = 1000, where *n* denotes the number of randomly permuted samples) from the copula family are shown using gray circles. The subplot title gives the country name followed by the stream and tide gauge in the following order: (**a**) United Kingdom (UK): Ouse – Immingham (**b**) France (FR): Oise – Dunkirk (**c**) Netherlands (NL): Rhine – Hoek van Holland (**d**) Germany (DE): Elbe – Cuxhaven (**e**) Sweden (SE): Göta älv - Gothenburg (**f**) Norway (NO): Austena – Oslo. The subscripts 'obs', 'sim', denote Kendall's τ correlation of observed data and simulated samples from the copula, respectively. The discrepancy between τ_{Sim} versus τ_{Obs} value in Hoek van Holland - Rhine, is attributed to the stronger UTDC exhibited by the compound event pair, which was satisfactorily modelled by Gumbel-Hougaard copula family as evidenced from small MESE statistics of CFG estimate (Table S6).

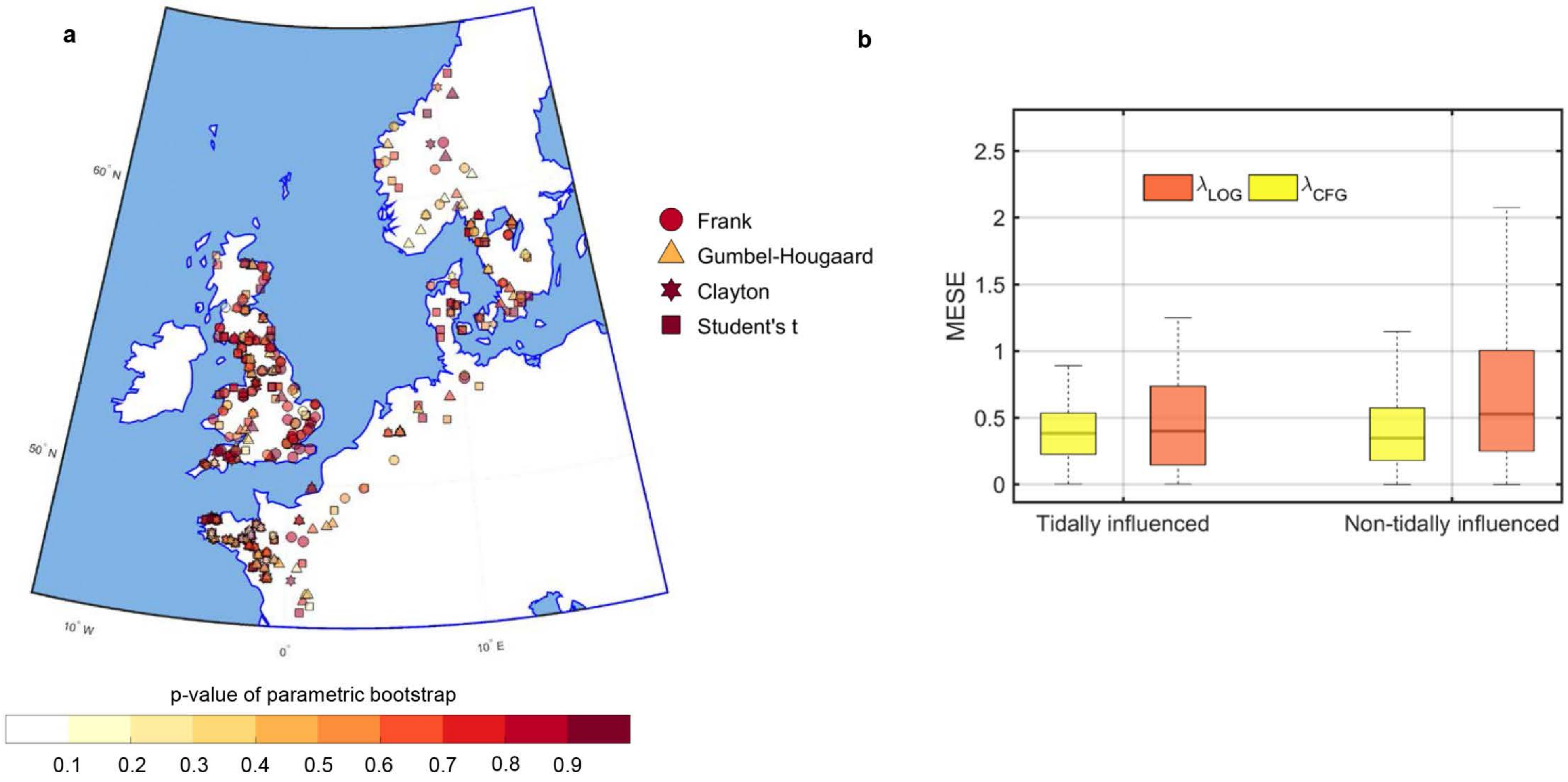

Figure S9. Goodness of fit tests for the selected copula families. (**a**) Locations of SGs; colour represents results of goodness of fit as measured by the p-value of the parametric bootstrap method, marker denotes the selected copula family. (**b**) Mean Error to Standard Error (MESE) ratio of UTDC for the best-selected copula family for the tidally influenced and non-tidally influenced SGs. The horizontal line (in black) in the box plot indicates the median, whereas the vertical span of the boxes indicates the inter-quartile range. The upper and lower whisker extends to the maximum and minimum value.

Table S1. Standardized anomaly* values of AMWL of affected TGs during storm Capella

Country	Sites	Latitude	Longitude	AMWL (m)	Mean, $\overline{X}$ (m)	Standard deviation, *SD*	$\overline{X}$ - *SD*	$\overline{X}$ + *SD*	Standardized anomaly, *Z*
Germany	Cuxhaven	53.87	8.72	10.09	8.65	0.54	8.10	9.19	**2.66**
Denmark	Esbjerg	55.47	8.43	3.27	2.67	0.46	2.21	3.13	**1.30**
France	Dunkirk	51.05	2.37	6.98	6.75	0.23	6.52	6.99	0.97
The Netherlands	Delfzijl	53.33	6.93	4.32	3.36	0.54	2.82	3.90	**1.79**
	Denhelder	52.96	4.75	2.95	2.17	0.31	1.86	2.47	**2.55**
	Hoek van Holland	51.98	4.12	2.9	2.41	0.26	2.15	2.67	**1.86**
United Kingdom	Dover	51.12	1.32	7.8	7.45	0.24	7.20	7.69	**1.46**
	Heysham	54.03	-2.92	10.69	10.64	0.34	10.30	10.98	0.13
	Immingham	53.63	-0.19	8.47	8.04	0.27	7.76	8.31	**1.58**
	Lowestoft	52.47	1.75	4.18	3.58	0.33	3.25	3.91	**1.81**

**Standardized anomaly is calculated as the deviation of respective AMWL from its long-term (1970-2014) mean, divided by the climatological standard deviation (SD). The standardized anomaly values of AMWL that exceed 1 SD are marked in bold.*

Table S2. Standardized anomaly values of AMWL of affected TGs during storm Xynthia

Country	Sites	Latitude	Longitude	AMWL (m)	$\bar{X}$ (m)	*SD*	$\bar{X}$ - *SD*	$\bar{X}$ + *SD*	*Z*
France	Cherbourg	49.65	-1.63	7.09	7.02	0.12	6.90	7.14	0.59
	Dunkirk	51.05	2.37	6.90	6.75	0.23	6.52	6.99	0.66
	Le Conquet	48.36	-4.78	7.70	7.54	0.17	7.37	7.71	0.93
	Le Havre	49.48	0.106	8.81	8.61	0.19	8.42	8.80	**1.07**
	Port Tudy	47.64	-3.45	6.04	5.80	0.15	5.66	5.95	**1.63**
	Roscoff	48.72	-3.96	9.74	9.68	0.17	9.51	9.86	0.36
	Saint Gildas	47.14	-2.25	7.0	6.32	0.17	6.14	6.49	**3.95**
	La Rochelle	46.16	-1.22	8.0	6.78	0.30	6.49	7.09	**4.01**
United Kingdom	Heysham	54.03	-2.92	10.49	10.64	0.34	10.30	10.98	-0.43

Table S3. Standardized anomaly values of AMWL of affected TGs during storm Xaver

Country	Sites	Latitude	Longitude	AMWL (m)	$\bar{X}$ (m)	*SD*	$\bar{X}$ - *SD*	$\bar{X}$ + *SD*	*Z*
Germany	Cuxhaven	53.87	8.72	9.64	8.46	0.54	8.10	9.19	**1.83**
The Netherlands	Delfzijl	53.33	6.93	4.52	3.36	0.54	2.82	3.90	**2.17**
	Denhelder	52.96	4.75	2.48	2.17	0.31	1.86	2.48	**1.03**
	Hoek van Holland	51.98	4.12	2.85	2.41	0.26	2.15	2.68	**1.69**
Norway	Heimsjoe	63.42	9.10	3.52	3.66	0.17	3.50	3.83	-0.86
	Tregde	58.00	7.55	1.78	1.81	0.14	1.67	1.95	-0.17
Sweden	Gothenburg	57.68	11.79	1.18	1.02	0.20	0.82	1.22	0.81
	Ringhals	57.25	12.11	1.27	0.96	0.22	0.74	1.18	**1.41**
	Stenungsund	58.09	11.83	1.11	1.1	0.20	0.90	1.30	0.07
United Kingdom	Aberdeen	57.14	-2.07	5.14	4.94	0.13	4.81	5.08	**1.52**
	Dover	51.12	1.32	8.35	7.45	0.24	7.21	7.69	**3.75**
	Heysham	54.03	-2.92	10.96	10.64	0.34	10.30	10.98	0.92
	Immingham	53.63	-0.19	9.06	8.04	0.27	7.76	8.31	**3.75**
	Lowestoft	52.47	1.75	4.74	3.58	0.33	3.25	3.91	**3.49**
	North shields	55.00	-1.44	6.52	5.76	0.20	5.56	5.96	**3.81**
	Wick	58.44	-3.09	4.12	4.15	0.12	4.03	4.26	-0.16

Table S4. Marginal distribution fits of AMWL and corresponding peak discharge (Q_{peak}) for selected TG-SG pairs

Country	TG Site	TG Location		SG* Site	RG Location		AIC_c Statistics of AMWL				AIC_c Statistics of Q_{Peak}			
		Latitude	Longitude		Latitude	Longitude	GEV	Kernel	Gamma	Log normal	GEV	Kernel	Gamma	Log normal
United Kingdom	Immingham	53.63	-0.18	6605570 (Ouse)	53.99	-1.13	-315.4	**-343.8**	-274.03	-274.7	-271.4	**-287.9**	-286.1	-267.2
France	Dunkirk	51.05	2.37	6122141 (Oise)	49.55	2.99	-259.8	**-276.7**	-270.73	-268.7	-244.9	**-270.4**	-259.1	-240.5
The Netherlands	Hoek van Holland	51.97	4.12	6435060 (Rhine)	51.84	6.11	-293.1	**-307.3**	-280.4	-285.9	-294.5	**-304.0**	-277.3	-300.5
Germany	Cuxhaven	53.87	8.72	6340110 (Elbe)	53.23	10.89	-255.8	**-296.0**	-228.7	-231.0	-301.6	**-320.4**	-278.8	-304.8
Sweden	Gothenburg	57.68	11.79	6229500 (Göta älv)	58.35	12.37	-319.4	-322.5	-314.9	**-325.8**	**-330.4**	-302.1	-222.8	-213.0
Norway	Oslo	59.91	10.73	6731280 (Austena)	58.85	8.1	-307.9	**-325.3**	-324.5	-316.3	-309.9	**-335.0**	-314.9	-290.7

*TG and SG denote tide gauge and stream gauge information. In the fifth column, the numerals indicate GRDC station number, followed by the name of the river in brackets. AIC_c indicates Akaike Information Criterion corrected for small sample size.

Table S5. Goodness of fit statistics for the best performing copula families for selected TG-SG pairs

Country	TG Site	TG Location		SG* Site	RG Location		Selected Copula family	Copula dependence parameter*		GoF test statistics		
		Latitude	Longitude		Latitude	Longitude		(θ / r)	ϑ	S_n^e	S_n^{k*}	p_{val}
United Kingdom	Immingham	53.63	-0.18	6605570 (Ouse)	53.99	-1.13	Student's *t*	r = 0.194	5	0.023	0.045	0.81
France	Dunkirk	51.05	2.37	6122141 (Oise)	49.55	2.99	Frank	θ = 1.17	-	0.028	0.046	0.51
The Netherlands	Hoek van Holland	51.97	4.12	6435060 (Rhine)	51.84	6.11	Gumbel-Hougaard	θ = 1.10	-	0.027	0.055	0.54
Germany	Cuxhaven	53.87	8.72	6340110 (Elbe)	53.23	10.89	Student's *t*	r = 0.05	5	0.029	0.044	0.42
Sweden	Gothenburg	57.68	11.79	6229500 (Göta älv)	58.35	12.37	Gumbel-Hougaard	θ = 1.22	-	0.027	0.047	0.43
Norway	Oslo	59.91	10.73	6731280 (Austena)	58.85	8.1	Gumbel-Hougaard	θ = 1.27	-	0.034	0.045	0.20

*Dependence parameter for Archimedean class of copula is θ whereas parameters for elliptical class of copula are r and ϑ

Table S6. Observed vs best performing copula families (from Table S5) in simulating Upper tail dependence coefficients (UTDC) of compound events

Country	TG Site	TG Location		SG Site	SG Location		UTDC				MESE statistics	
		Latitude	Longitude		Latitude	Longitude	λ_{CFG}^{Obs}	$\mu\left(\lambda_{CFG}^{Sim}\right)^{**}$	λ_{LOG}^{Obs}	$\mu\left(\lambda_{LOG}^{Sim}\right)$	ε_{CFG}	ε_{LOG}
United Kingdom	Immingham	53.63	-0.18	6605570 (Ouse)	53.99	-1.13	0.174 (0.048)	0.237	0.276 (0.0235)	0.264	0.576	0.088
France	Dunkirk	51.05	2.37	6122141 (Oise)	49.55	2.99	0.19 (0.0314)	0.30	0.22 (0.0895)	0.27	0.35	0.24
The Netherlands	Hoek van Holland	51.97	4.12	6435060 (Rhine)	51.84	6.11	0.148 (0.150)	0.211	0.346 (0.0424)	0.27	0.55	0.48
Germany	Cuxhaven	53.87	8.72	6340110 (Elbe)	53.23	10.89	0.097 (0.165)	0.156	0.296 (0.074)	0.21	0.53	0.56
Sweden	Gothenburg	57.68	11.79	6229500 (Göta älv)	58.35	12.37	0.29 (0.0058)	0.30	0.49 (0.0008)	0.36	0.07	0.85
Norway	Oslo	59.91	10.73	6731280 (Austena)	58.85	8.1	0.31 (0.0187)	0.32	0.14 (0.0487)	0.37	0.14	1.57

Note: λ_{CFG}^{Obs} *and* λ_{LOG}^{Obs} *denote empirical UTDC estimates using CFG and LOG estimators; MESE indicates mean error to standard error statistics* (*See Methods*). The *p-values* of observed *UTDCs* are enclosed within brackets and obtained from *N* = 10,000 bootstrap simulations. $\mu(\bullet)^{**}$ *denotes mean UTDC statistics obtain from N = 500 simulated samples from the selected parametric family of copula.*

.

Supplementary References

1. Moftakhari, H. R., Salvadori, G., AghaKouchak, A., Sanders, B. F. & Matthew, R. A. Compounding effects of sea level rise and fluvial flooding. *Proceedings of the National Academy of Sciences* **114**, 9785–9790 (2017).
2. Moon, Y.-I. & Lall, U. Kernel quantite function estimator for flood frequency analysis. *Water Resources Research* **30**, 3095–3103 (1994).
3. Wahl, T., Jain, S., Bender, J., Meyers, S. D. & Luther, M. E. Increasing risk of compound flooding from storm surge and rainfall for major US cities. *Nature Climate Change* **5**, 1093–1097 (2015).
4. Hurvich, C. M. & Tsai, C.-L. Model selection for extended quasi-likelihood models in small samples. *Biometrics* 1077–1084 (1995).
5. Gringorten, I. I. A plotting rule for extreme probability paper. *Journal of Geophysical Research* **68**, 813–814 (1963).
6. Zhang L. & Singh V. P. Bivariate Flood Frequency Analysis Using the Copula Method. *Journal of Hydrologic Engineering* **11**, 150–164 (2006).
7. Frahm, G., Junker, M. & Schmidt, R. Estimating the tail-dependence coefficient: properties and pitfalls. *Insurance: mathematics and Economics* **37**, 80–100 (2005).
